# *In Vivo* Structure of the *E. coli* FtsZ-ring Revealed by Photoactivated Localization Microscopy (PALM)

**DOI:** 10.1371/journal.pone.0012680

**Published:** 2010-09-13

**Authors:** Guo Fu, Tao Huang, Jackson Buss, Carla Coltharp, Zach Hensel, Jie Xiao

**Affiliations:** Department of Biophysics and Biophysical Chemistry, Johns Hopkins University School of Medicine, Baltimore, Maryland, United States of America; Texas A&M University, United States of America

## Abstract

The FtsZ protein, a tubulin-like GTPase, plays a pivotal role in prokaryotic cell division. *In vivo* it localizes to the midcell and assembles into a ring-like structure-the Z-ring. The Z-ring serves as an essential scaffold to recruit all other division proteins and generates contractile force for cytokinesis, but its supramolecular structure remains unknown. Electron microscopy (EM) has been unsuccessful in detecting the Z-ring due to the dense cytoplasm of bacterial cells, and conventional fluorescence light microscopy (FLM) has only provided images with limited spatial resolution (200–300 nm) due to the diffraction of light. Hence, given the small sizes of bacteria cells, identifying the *in vivo* structure of the Z-ring presents a substantial challenge. Here, we used photoactivated localization microscopy (PALM), a single molecule-based super-resolution imaging technique, to characterize the *in vivo* structure of the Z-ring in *E. coli*. We achieved a spatial resolution of ∼35 nm and discovered that in addition to the expected ring-like conformation, the Z-ring of *E. coli* adopts a novel compressed helical conformation with variable helical length and pitch. We measured the thickness of the Z-ring to be ∼110 nm and the packing density of FtsZ molecules inside the Z-ring to be greater than what is expected for a single-layered flat ribbon configuration. Our results strongly suggest that the Z-ring is composed of a loose bundle of FtsZ protofilaments that randomly overlap with each other in both longitudinal and radial directions of the cell. Our results provide significant insight into the spatial organization of the Z-ring and open the door for further investigations of structure-function relationships and cell cycle-dependent regulation of the Z-ring.

## Introduction

The FtsZ protein is a highly conserved tubulin-like GTPase that plays an essential role in bacterial cell division [1]–[3]. *In vitro*, FtsZ assembles into single-stranded protofilaments and higher-order structures such as ribbons, sheets and bundles [4]–[6]. *In vivo*, FtsZ assembles into a supramolecular ring-like structure (hence named the Z-ring) at the midcell to drive cell division [7]. The structures of FtsZ polymers *in vitro* have been extensively characterized using EM, but the structure of the Z-ring *in vivo* has remained elusive.

Knowing the *in vivo* structure of the Z-ring will contribute significantly to the molecular understanding of the scaffolding and constricting functions of the Z-ring. Despite this well-appreciated importance, past attempts to determine the structure of the Z-ring have been unsuccessful. Using conventional fluorescence light microscopy (FLM), the Z-ring appears to adopt a smooth, closed circular conformation [8], [9]. Under some circumstances FtsZ outside of the midcell Z-ring in *B. subtilis* was found to form highly dynamic helical structures extending from the midcell to the cell pole [10], [11]. Similar but much less distinct structures have also been observed in wild-type (wt) *E. coli* cells using immunofluorescence [12] and in cells overexpressing a fluorescent protein (FP) fusion of FtsZ [13]. These observations led to the hypothesis that the Z-ring itself may result from the compression of an extended helical structure [10]. However, because of the limited spatial resolution of FLM (200–300 nm) [14], this hypothesis has not been verified.

It is well accepted that the Z-ring consists of an arrangement of short FtsZ protofilaments averaging 30 subunits long [15]–[17] and that these protofilaments are tethered to the membrane by two membrane-binding proteins, FtsA and ZipA [18], [19]. However, the arrangement of protofilaments inside the Z-ring is unknown. EM images of bacterial cytoplasm do not show the presence of the Z-ring, possibly because the cytoplasm is too dense to offer good contrast, or because protofilaments inside the Z-ring are not sufficiently ordered to appear distinguishable [20], [21]. ImmunoEM has also been unsuccessful because the antibody labeling density is too low to reveal any distinct structural features of the Z-ring [7]. Only one recent study using electron cryotomography (ECT) successfully detected single filaments in *C. crescentus* that were believed to be FtsZ protofilaments [22]. This study showed that instead of a continuous ring structure, the Z-ring of *C. crescentus* is composed of two or three protofilaments scattered randomly along the membrane at the midcell. However, the low number of protofilaments observed in this study is difficult to reconcile with the expected number (20–80 protofilaments) calculated from the concentration and midcell localization percentage of FtsZ in *C. crescentus* [23].

To overcome the above limitations, in this work we took advantage of the newly developed super-resolution imaging method PALM to elucidate the *in vivo* structure of the *E. coli* Z-ring [24]. PALM imaging combines the advantages of both conventional EM and FLM by providing spatial resolutions in the nanometer range while at the same time allowing unambiguous identification of a cellular structure through specific fluorescent labeling [24], [25]. We labeled FtsZ with the newly developed photoactivatable FP mEos2 [26] and were able to image the Z-ring with ∼35 nm spatial resolution, an order of magnitude improvement over the spatial resolution of conventional FLM. We discovered that in addition to a ring conformation, the Z-ring of *E. coli* adopts a variety of compressed helical conformations with tight pitches ranging from 50–400 nm. Next, we measured the width and density of the Z-ring, which describe how many protofilaments-thick the ring is and how many FtsZ molecules are packed in a unit area. These structural details have not been available before and are difficult or impossible to obtain by other means. Finally, we examined the dependence of the conformation, width and density of the Z-ring on the total cellular FtsZ level. Based on our results, we propose that the Z-ring of *E. coli* is composed of a loose bundle of FtsZ protofilaments that randomly overlap with each other in both longitudinal and radial directions of the cell. This arrangement of protofilaments is different from the single-layered flat ribbon or random arrangement of protofilaments suggested previously [4], [15], [22], [27].

## Results

### FtsZ-mEos2 is a reliable label for PALM imaging of Z-ring structure *in vivo*

We chose mEos2 for PALM imaging because of its superior properties: mEos2 folds well, is minimally disruptive for tightly packed proteins and has a high contrast ratio before and after photoactivation [26], [28]. In addition, mEos2 emits green fluorescence before photoactivation, which allows the ensemble appearance of the Z-ring to be recorded before PALM imaging, enabling direct comparison between the conventional and subdiffraction-limited images of the Z-ring.

We fused mEos2 to the C-terminus of FtsZ and examined whether the fusion protein was able to replace endogenous wt FtsZ. As with all other known FtsZ-FP fusions, FtsZ-mEos2 did not rescue the lethal phenotype of the conditional *ftsZ* deletion strain JKD7-2/pKD3a at nonpermissive temperature. We have screened different linker sequences between FtsZ and mEos2 and placed mEos2 at the N- or C-terminus as well as internal positions of FtsZ, but none of these fusion proteins complemented the JKD7-2/pKD3a strain.

Although the fusion protein cannot function as the sole cellular source of FtsZ, based on two previous studies we reason that the fusion of mEos2 to FtsZ may have minimal impact on the structure of the Z-ring. In one study, a membrane-targeting sequence (mts) was placed at the C-terminus of an FtsZ-YFP fusion. The resulting fusion protein FtsZ-YFP-mts was able to self-assemble into ring-like structures in the JKD7-2/pKD3a strain at a nonpermissive temperature and contract in the presence of GTP in a reconstituted liposome system [29]. This study showed that the fusion of a FP moiety to FtsZ does not disrupt the ability of FtsZ to assemble into a contractile Z-ring once a membrane anchor is provided. It also hinted that the reason FtsZ-FP fusion proteins fail to support cell division is likely due to an inability to bind the membrane anchoring proteins, FtsA and ZipA, which are required for the recruitment of downstream division proteins to complete divisome assembly [30]. In a second study, the morphologies of single protofilaments and sheets of protofilaments made of wt FtsZ and the FtsZ-YFP fusion protein were found to be indistinguishable from each other using EM [31]. This result suggests that the fusion of an FP to the C-terminus of FtsZ does not impose detectable perturbations to the resulting higher-order structures of FtsZ even on the nanometer scale. Therefore, it is reasonable to expect that the FtsZ-mEos2 fusion protein would allow the normal assembly of the Z-ring and serve as a reliable structural label for the Z-ring.

**Figure 1:**
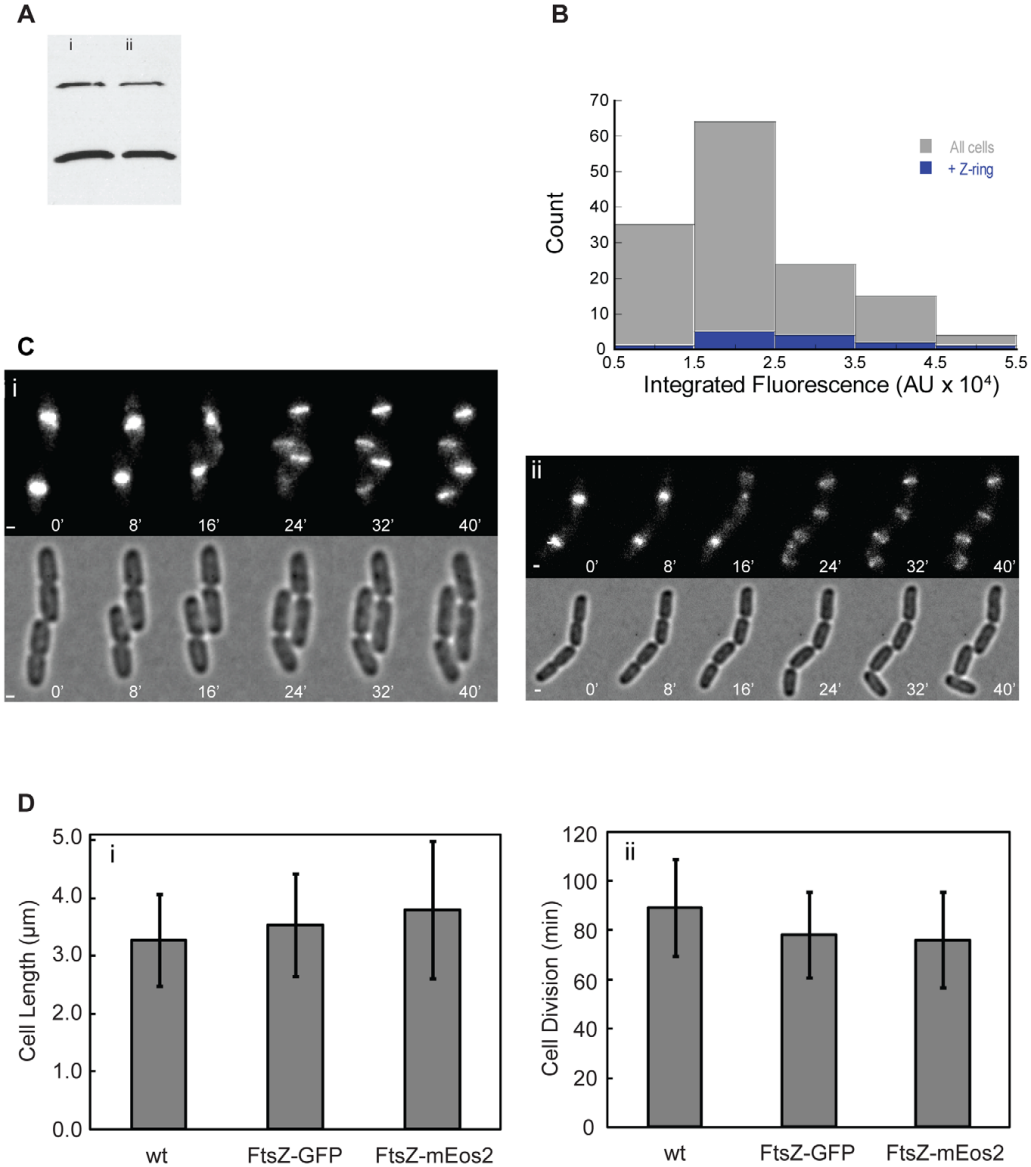
Characterization of expression and function of FtsZ and FtsZ-mEos2. A. Representative immunoblot used to quantify the expression level of FtsZ-mEos2 (top) relative to that of wt FtsZ (bottom) in BL21(DE3)pLysS cells grown under the described condition used for PALM. On average FtsZ-mEos2 is expressed at ∼25% of total intracellular FtsZ concentration. Lanes i and ii illustrate dilutions of the same sample. Duplicate gels were analyzed. B. Histograms of the total integrated green-channel fluorescence of BL21(DE3)pLysS cells expressing FtsZ-mEos2 from pET28 under the induction condition used for PALM. The mean (2.2×10^4^±1.0×10^4^) and distribution (gray) of the entire population are not significantly different from the mean (2.6×10^4^±1.2×10^4^) and distribution (blue) of the population containing Z-rings. C. Live-cell time-lapse image montages of *E. coli* B/r A cells expressing FtsZ-mEos2 (i) or FtsZ-GFP (ii) are displayed with epi-fluorescence green-channel images atop corresponding bright-field images. Both FtsZ-FPs localize to midcell around the same time (in minutes) after the previous round of cell division. D. Average cell lengths (i) for B/r A cells expressing FtsZ-GFP (N = 438), FtsZ-mEos2 (N = 332) or no fusion proteins (N = 263) and the average cell doubling time (ii) measured from time-lapse imaging of B/r A cells expressing FtsZ-GFP (N = 55), FtsZ-mEos2 (N = 50) or no fusion proteins (N = 27). Bars, 1000 nm.

To verify the above expectation, we first examined expression levels of FtsZ and FtsZ-mEos2 in the strain BL21 (DE3) pLysS, which was used for PALM imaging. Using immunoblotting we found that on average the strain expresses 4100±1700 FtsZ molecules/cell under the same growth condition used for PALM imaging. This average expression level is consistent with previous reports (3000–5000 molecules/cell) [32], [33], but lower than the 10,000–15,000 molecules/cell reported by Lu et al [34]. The fusion protein FtsZ-mEos2 was expressed at approximately 25% of total cellular FtsZ (wt FtsZ + FtsZ-mEos2) at the induction level used for PALM imaging (Figure 1A). Previous studies have shown that up to 50% of total cellular FtsZ could be FtsZ-FP fusion without detectable growth defects [8], [12], [13], [35].

Because the BL21 (DE3) pLysS strain uses the strong T7 promoter to express FtsZ-mEos2, the induction, although brief and in the presence of the pLysS plasmid, could have created a population with heterogeneous expression levels. Some cells may express extremely high levels while other express very low levels of FtsZ-mEos2. Consequently, the selection of cells used for PALM imaging (those exhibiting midcell FtsZ-mEos2 localization) may have different expression distributions from the whole cell population.

To examine this possibility, we measured the integrated green fluorescence, which reflects the expression level of FtsZ-mEos2, in individual cells of the BL21(DE3) pLysS strain on a fluorescence microscope. We found that the integrated fluorescence intensity of individual cells largely followed a normal distribution (Figure 1B). The coefficient of variation (CV, standard deviation/mean) is 0.44. A CV value of lower than one, which is expected for an exponential distribution, is usually considered to be of low variability. Most importantly, cells showing midcell Z-ring localization have a similar intensity distribution and mean intensity to that of the whole cell population (Figure 1B). These observations suggest that the selection of cells showing midcell Z-ring localization for PALM imaging is not biased toward cells expressing extremely high FtsZ-mEos2 levels.

Having quantified the expression level of FtsZ-mEos2 and wt FtsZ, we examined whether FtsZ-mEos2 was able to localize to the midcell in the presence of wt FtsZ in a cell cycle-dependent manner. This is the standard used in the field to determine if an FtsZ-FP fusion protein retains its ability to associate with wt FtsZ and serves as an accurate label for FtsZ localization and dynamics [8], [12], [13], [15], [30], [35], [36]. We then compared this result with that of an FtsZ-GFP fusion, since FtsZ-GFP fusions were shown to have an indistinguishable localization pattern when compared to that of wt FtsZ using immunofluorescence [13], [37].

To ensure a proper comparison, we replaced the *ftsZ-gfp* gene in the expression vector (multi-copy plasmid pCA24N with an inducible *lac* promoter [38]) with the *ftsZ-mEos2* gene and expressed the FtsZ-mEos2 fusion protein in the same B/r A strain under the same growth condition as the FtsZ-GFP fusion. We found that in the presence of wt FtsZ, FtsZ-mEos2 correctly localized to midcell in a cell cycle-dependent manner, similar to that of the FtsZ-GFP fusion (Figure 1C). In exponentially growing cultures, 80% of cells showed FtsZ-mEos2 midcell localization, which is comparable to that of FtsZ-GFP (85%). When wt FtsZ was depleted, cells expressing FtsZ-mEos2 showed diffusive fluorescence instead of clear Z-rings at midcell, demonstrating that the midcell localization of FtsZ-mEos2 is wt FtsZ dependent. In addition, cells expressing FtsZ-mEos2 showed similar cell length and doubling time as those of cells expressing FtsZ-GFP and of parent strain cells expressing no FtsZ-FP fusion protein (Figure 1D).

The above experiments established that FtsZ-mEos2 incorporates into the Z-ring through the association with wt FtsZ, is expressed homogenously across the cell population, and does not impair cell division at the expression level used for PALM imaging. Based on these results we conclude that FtsZ-mEos2 has a minimal impact on the *in vivo* structure of the Z-ring and can be used to label the Z-ring for PALM imaging.

### The midcell Z-ring adopts a variety of helical conformations in addition to a ring-like conformation

Previous studies have shown that the Z-ring is highly dynamic [15]–[17]. Extended FtsZ helical structures have been observed to constantly collapse or emanate from the midcell Z-ring [10]–[12]. Therefore, in order to obtain a clear snapshot of the Z-ring, we fixed cells of the strain BL21(DE3)pLysS and performed PALM imaging on cells that had a normal length and showed a single green fluorescent band at midcell.

**Figure 2:**
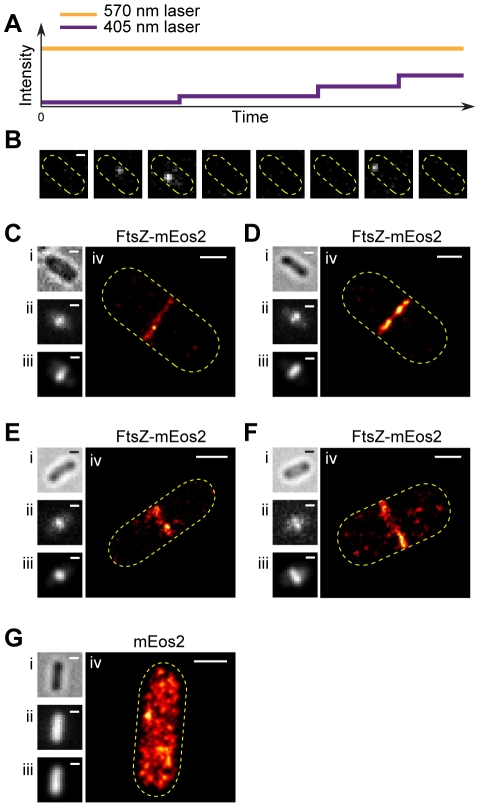
PALM imaging of the Z-ring in *E. coli* cells. A. Schematic representation of photoactivated localization microscopy (PALM) imaging sequence using continuous illumination of the 570-nm excitation laser (yellow line) and the 405-nm activation laser (purple line) simultaneously. The illumination intensity of the 570-nm excitation laser was kept constant throughout the imaging sequence while that of the 405-nm activation laser was increased stepwise as the pool of inactivated FtsZ-mEos2 molecules was depleted over time. B. Fluorescent images of single FtsZ-mEos2 molecules in one *E. coli* cell excited by the 570-nm laser during one typical PALM imaging sequence. Usually a single FtsZ-mEos2 molecule was detected and photobleached in one single frame (frames 2, 3 and 7). Bar, 500 nm. C. Images of cells expressing FtsZ-mEos2 and mEos2 in the order of bright-field (i), ensemble green fluorescence (ii), regenerated ensemble fluorescence image (iii) and PALM images (iv). The bright-field image of a cell was first taken, and then the ensemble fluorescence image of the cell in the green channel was taken. The PALM imaging sequence was then initiated to record tens of thousand frames similar to what was shown in B. The corresponding PALM image was then constructed from the image stack (Text S1). The regenerated ensemble fluorescence images were constructed by expanding the point-spread-function (PSF) of each single mEos2 molecule in the PALM image to a diffraction-limited spot. These images were indistinguishable from the original epi-fluorescence images, validating the PALM image construction algorithm. The number of molecules used to construct the PALM images were 1083 (C), 1109 (D), 205 (E), 359 (F), and 2277 (G), respectively. Bars, 500 nm.

We first recorded the contour of the cell in the bright-field and the ensemble appearance of the Z-ring in the green fluorescence channel prior to PALM imaging (Figure 2C). We then used a near-UV laser (405 nm) simultaneously with a yellow laser (570 nm) in epi-illumination mode to continuously activate and excite single FtsZ-mEos2 molecules (Figure 2A). The excitation power of the 570-nm laser was adjusted high enough so that most molecules were imaged and photobleached within one frame (Figure 2B). Acquisition continued until 20,000 frames were acquired or until FtsZ-mEos2 activation became very infrequent. The positions of single FtsZ-mEos2 molecules in each frame of this series were then determined, calibrated and plotted together to construct a PALM image (Figure 2C and Text S1). Only molecules with a localization precision of 25 nm or better were used to construct PALM images. The average localization precision of plotted FtsZ-mEos2 molecules in images reported in this work was 14±6 nm unless otherwise noted (Text S1, Figure S1A). The average spatial resolution of the resulting PALM images, determined by calculating the displacement between the fitted positions of the same molecule in two consecutive frames, was 34±20 nm (see below, Text S1 and Figure S1C).

Figures 2C–2F show the PALM images of four typical BL21(DE3)pLysS cells expressing FtsZ-mEos2. As expected for a circular Z-ring that is perpendicular to the long axis of the cell, we observed a thin, sharp band at the midcell. This observation is distinctly different from the diffusive cytoplasmic localization of mEos2 by itself (Figure 2G), indicating that the localization pattern we observed for FtsZ-mEos2 was specific to FtsZ.

**Figure 3:**
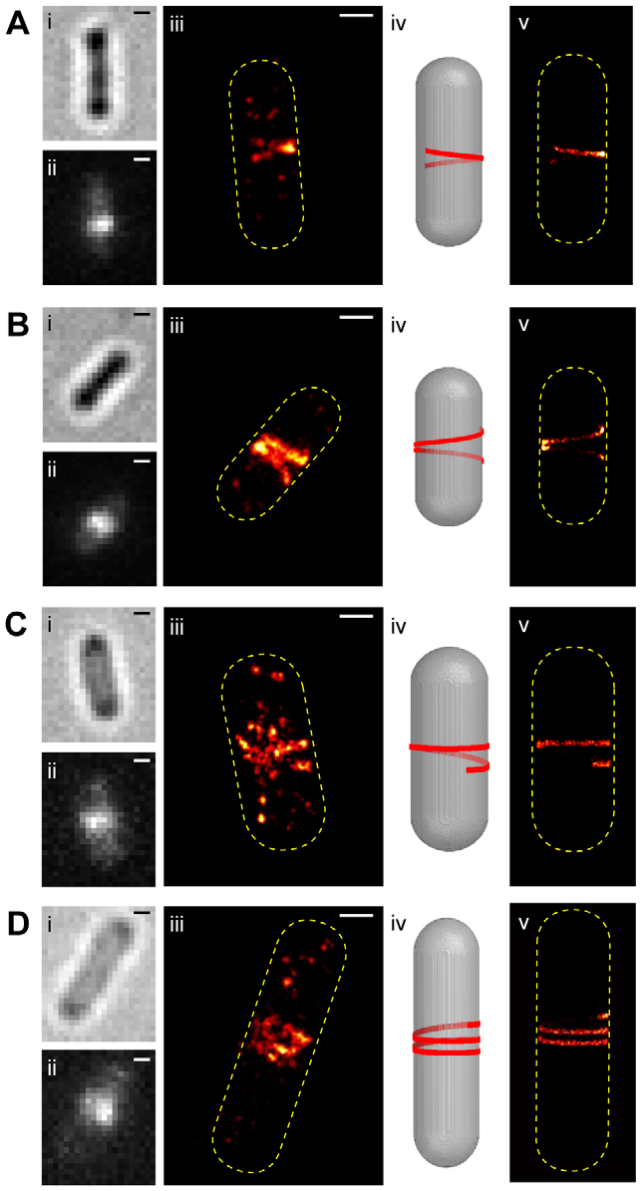
PALM images of representative fixed *E. coli* BL21(DE3)pLysS cells expressing FtsZ-mEos2. Images of fixed cells expressing FtsZ-mEos2 in the order of bright-field (i), ensemble green fluorescence prior to PALM imaging (ii), PALM image (iii), models of helical structures in 3D *E. coli* cells (iv, Text S2), and simulated PALM images resulting from the helical models shown in iii (v, Text S2). The numbers of FtsZ-mEos2 molecules used to construct the PALM images were 999 (A), 2843 (B), 804 (C), and 627 (D), respectively. Bars, 500 nm.

In addition to a single sharp band at the midcell, we also observed that the single ensemble green channel fluorescent band of some cells resolved into two or more closely-spaced thin bands in the corresponding PALM images (Figure 3, column iii). Interestingly, these thin PALM bands appeared to adopt a variety of conformations with different lengths, tilting angles and separation distances ranging from 50–400 nm.

Since PALM images are 2-dimensional (2D) projections of 3-dimensional (3D) structures inside the cells, based on visual inspection we suspected that the observed patterns of multiple bands resulted from 3D helical structures along the inner membrane surfaces. To verify this possibility, we modeled an FtsZ helix along the inner cylindrical surface of a typical *E. coli* cell using a set of measured parameters including the length, tilting angles and positions of the thin bands from the PALM images, the size of an FtsZ monomer and the number of total FtsZ molecules inside the cell (Figure 3, column iv, Text S2 and Figure S2). We then projected this helix onto a 2D plane and produced a simulated PALM image (Figure 3, column v) by taking into account multiple factors that would influence the final PALM image such as the defocusing effect, stochastic activation of FtsZ-mEos2 molecules and single molecule photon fluctuations (Text S2).

We found that the best way to produce a simulated PALM image that matched the measured tilting angles of bands and the separation distances between bands in the real PALM images was by modeling a helix with variable pitches along the helical path (Text S2). If a helix with a constant pitch is modeled and projected onto a 2D plane, the distance between the simulated PALM bands, which is determined by the pitch, is significantly different from the measured distance between the real PALM bands (Text S2 and Table S1). Therefore, we allowed the pitch along the helical path to vary and successfully simulated PALM images that matched well with the real PALM images (Figure 3, column iv, and Figure S2). In addition, the lengths of these helices varied from cell to cell, further establishing the large variability of these helical conformations.

### Compressed helical structures are not artifacts caused by chemical fixation

Chemical fixation may distort cellular structures at the nanometer scale, especially when the structure of interest is attached to the membrane [39]. To avoid chemical fixation artifacts or any other possible structural alterations during sample preparation, it is best to image the Z-ring in live cells. However, currently most PALM imaging is conducted on fixed cells because it usually takes several minutes to acquire the thousands of image frames required for a single PALM image of a reasonable spatial resolution. Although live-cell PALM imaging has been realized before, it was conducted on cellular structures with slow dynamics [40], [41]. Based on fluorescence recovery after photobleaching (FRAP) experiments we know that the Z-ring turns over on a fast time scale of 10–30 s [15], [16]. Therefore, in order to obtain a clear PALM image of the Z-ring in live cells, it is critical to speed up the PALM imaging acquisition time.

**Figure 4:**
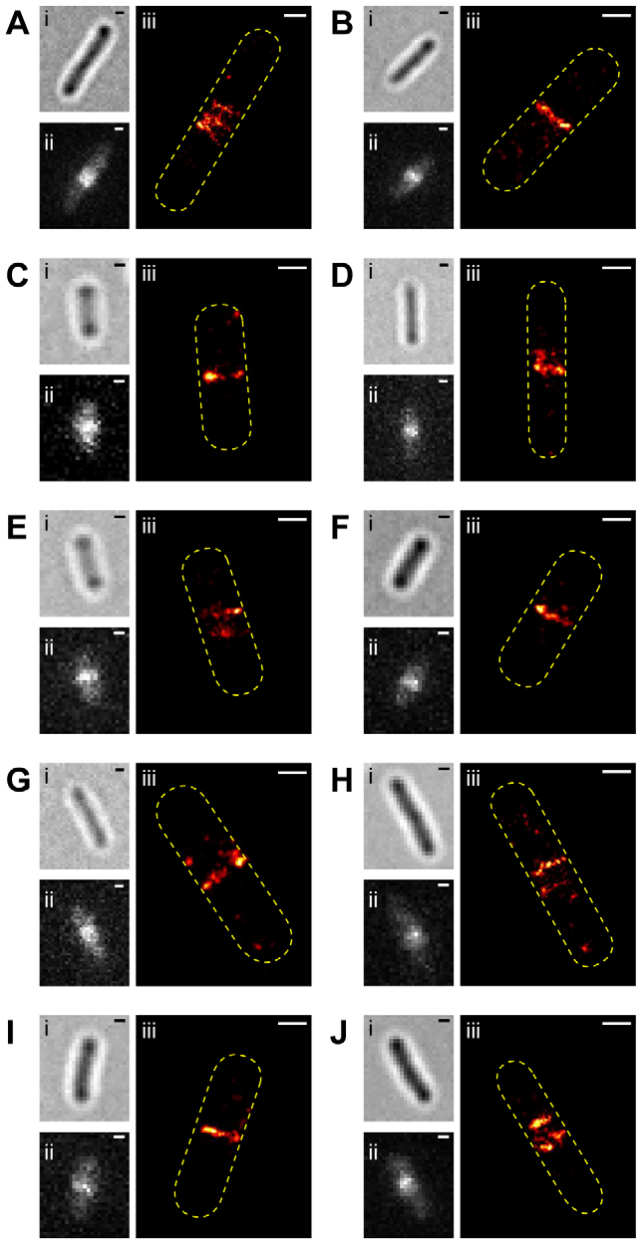
PALM images of representative live *E. coli* BL21(DE3)pLysS cells expressing FtsZ-mEos2. Images of live cells expressing FtsZ-mEos2 in the order of bright-field (i), ensemble green fluorescence prior to PALM imaging (ii), and PALM image (iii), The numbers of FtsZ-mEos2 molecules used to construct the PALM images were 301 (A), 185 (B), 165 (C), 187 (D), 265 (E), 224 (F), 155 (G), 187 (H), 222 (I), and 268 (J), respectively. Bars, 500 nm.

To image the Z-ring in live *E. coli* cells with a high spatial resolution, we developed a fast PALM imaging protocol. In this protocol we sped up the imaging time by using a high excitation intensity (1.8–2.5 kW/cm^2^), short exposure time (7 ms) and small illumination area (50×50 pixels). We were able to acquire a live-cell PALM image of the Z-ring within 20 s with a similar localization precision to the fixed cells images (23±7 nm). This 20-s imaging time is fast enough to “freeze” the Z-ring in action. Figure 4 shows 10 representative live-cell PALM images of BL21(DE3)pLysS cells expressing FtsZ-mEos2 under the same growth conditions used in fixed-cell PALM imaging. As with the fixed cells, some live cells showed one single PALM band at the midcell and some cells showed two or three thin PALM bands reminiscent of a helical structure inside the cell. This result strongly suggests that the helical conformation of the Z-ring we observed above is not an artifact caused by fixation but a genuine structural feature of the Z-ring.

### The Z-ring is a few protofilaments thick

To gain structural insight into the arrangement of FtsZ protofilaments inside the Z-ring, we measured the width of each individual PALM band using images obtained on fixed cells. We found that the band widths were similar to each other with an average of 113±25 nm (N = 17, Figure S3). The average band width in live cells (110±21 nm, N = 20) was also indistinguishable from that measured with fixed cells (p = 0.64).

To find out whether the similar band widths resulted from limited spatial resolution, we performed the following analyses. First, we calculated the theoretical spatial resolution based on two methods. Using the mean localization precision of 14 nm, we estimated that a single protofilament would appear to be 33 nm wide in our PALM images (Text S1). Therefore, if two protofilaments are spaced 33 nm apart on a projected 2D image plane we will be able to resolve them individually. This number is the upper limit of our achievable spatial resolution. In a second method, we calculated the spatial resolution according to the Nyquist criterion, which states that to achieve a desired spatial resolution of *n* nm, the sampling frequency should be at least *n*/2 nm [41]. In other words, a 10-nm spatial resolution requires that there is one labeled molecule every 5 nm along the structure. At a low labeling percentage of 25%, we calculated that on average the Nyquist spatial resolution is 28 nm (Text S1). This value is comparable to that determined from the average localization precision, indicating that the theoretical spatial resolution is not limited by the labeling percentage of the Z-ring. Next, we determined the actual measurement error by isolating molecules that lasted more than one frame in the PALM imaging sequence and plotting the apparent displacement between subsequent frames of these molecules (Text S1, Figure S1C, note that only the first frames of these molecules were used to construct the PALM images). The distribution of displacement fit well to a normal distribution with a main peak at 34 nm, in agreement with the upper limit of spatial resolution calculated based on the localization precision. Therefore, we estimate that the actual spatial resolution in our PALM images is ∼35 nm, which is still significantly smaller than the measured band width at 110 nm.

Because the average band width was ∼110 nm, much wider than the apparent width of a single protofilament in PALM images, the Z-ring must contain more than one protofilament laterally. This observation is consistent with previous estimations based on the average number of FtsZ monomers inside the cell [15], [20]. However, it is not yet possible to calculate precisely how many protofilaments-thick the Z-ring is based on the measured band width with 35-nm spatial resolution.

### The Z-ring is composed of overlapping FtsZ protofilaments

Knowing how tightly FtsZ molecules are packed inside the Z-ring will lend insight into the spatial arrangement of protofilaments. For a flat ribbon in which FtsZ protofilaments are spaced 9-nm apart [22] in a single layer, we calculated that the packing would be maximally 5 FtsZ monomers per PALM pixel (15×15 nm^2^) in the PALM image (1/(5×9) = 0.022 molecules/nm^2^, or 5 molecules/PALM pixel). By comparing the number of individual FtsZ-mEos2 molecules detected per pixel in the PALM bands to the theoretical number estimated from the flat ribbon configuration, we can assess if such a configuration is possible. However, because a PALM image is the 2D projection of a 3D structure, FtsZ molecules in the cytoplasm and on both surfaces of the cell will contribute differently to the projected image depending on where the focal plane is, leading to an inaccurate estimation of FtsZ packing densities in the corresponding PALM image.

**Figure 5:**
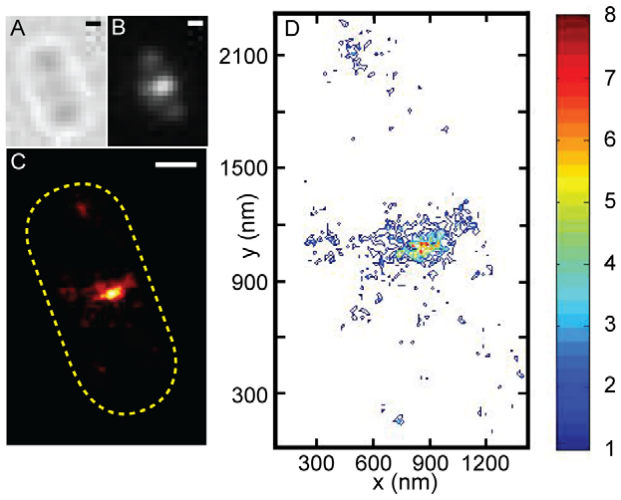
TIR PALM image of an *E. coli* BL21(DE3)pLysS cell expressing FtsZ-mEos2. Representative image of a fixed cell expressing FtsZ-mEos2 in the order of bright-field (A), total internal reflection (TIR) ensemble fluorescence image of the cell prior to TIR PALM imaging (B), TIR PALM image (C), and contour plot of FtsZ packing density in units of number of FtsZ-mEos2 molecules per PALM pixel (15×15 nm^2^). The number of FtsZ-mEos2 molecules used to construct the PALM image was 1704. Bars, 500 nm.

To circumvent this problem, we performed total internal reflection (TIR) PALM imaging. By adjusting the incident angle of the activation and excitation lasers, TIR allowed detection of FtsZ-mEos2 molecules in a thin layer (∼200 nm) at the cell-glass interface. Therefore, only FtsZ-mEos2 molecules that are located on the bottom membrane of the cell could be selectively excited and imaged. Figure 5C shows a typical TIR PALM image of one cell with a single band at midcell. The intensity of the band in the middle was higher than that on the two edges; this is the opposite of what we observed using epi-illumination as shown in Figures 2, 3 and 4, which is expected for the curved surface of a rod-shaped cell under TIR illumination.

We counted the number of FtsZ-mEos2 molecules per pixel in the TIR PALM images and found that approximately 40% of the pixels in the midcell band have a density ranging from two to eight FtsZ-mEos2 molecules per pixel (Figure 5D). Similar results were found in other TIR PALM images of different cells. We note that the density distribution of the midcell band is not uniform, which may indicate that FtsZ protofilaments inside the Z-ring are not uniformly distributed. However, it is also possible that the non-uniform density distribution is due to the stochastic activation of FtsZ-mEos2 molecules. Considering the facts that the expected maximal density for a single-layered FtsZ protofilament arrangement is about 5 FtsZ molecules/PALM pixel, that not all FtsZ molecules inside the Z-ring were labeled with mEos2, and that not all mEos2 molecules are fluorescent due to the slow maturation process of the fluorophore [26], the observation of large regions with high densities suggests that some regions of the Z-ring are composed of more than one layer of FtsZ protofilaments that overlap with each other along the radial direction of the cell.

### Concentration dependence of Z-ring structure

It is known that the assembly of FtsZ superstructures *in vitro* is concentration-dependent [1], [3], and that overexpression of FtsZ *in vivo* often leads to multiple Z-rings and spiral structures [13]. However, because of limited spatial resolution in conventional FLM, it is difficult to assess in detail how the structure of the Z-ring responds to different FtsZ expression levels. Here we examine the effect of cellular FtsZ levels on the conformation, width and density of the Z-ring using PALM imaging.

**Figure 6:**
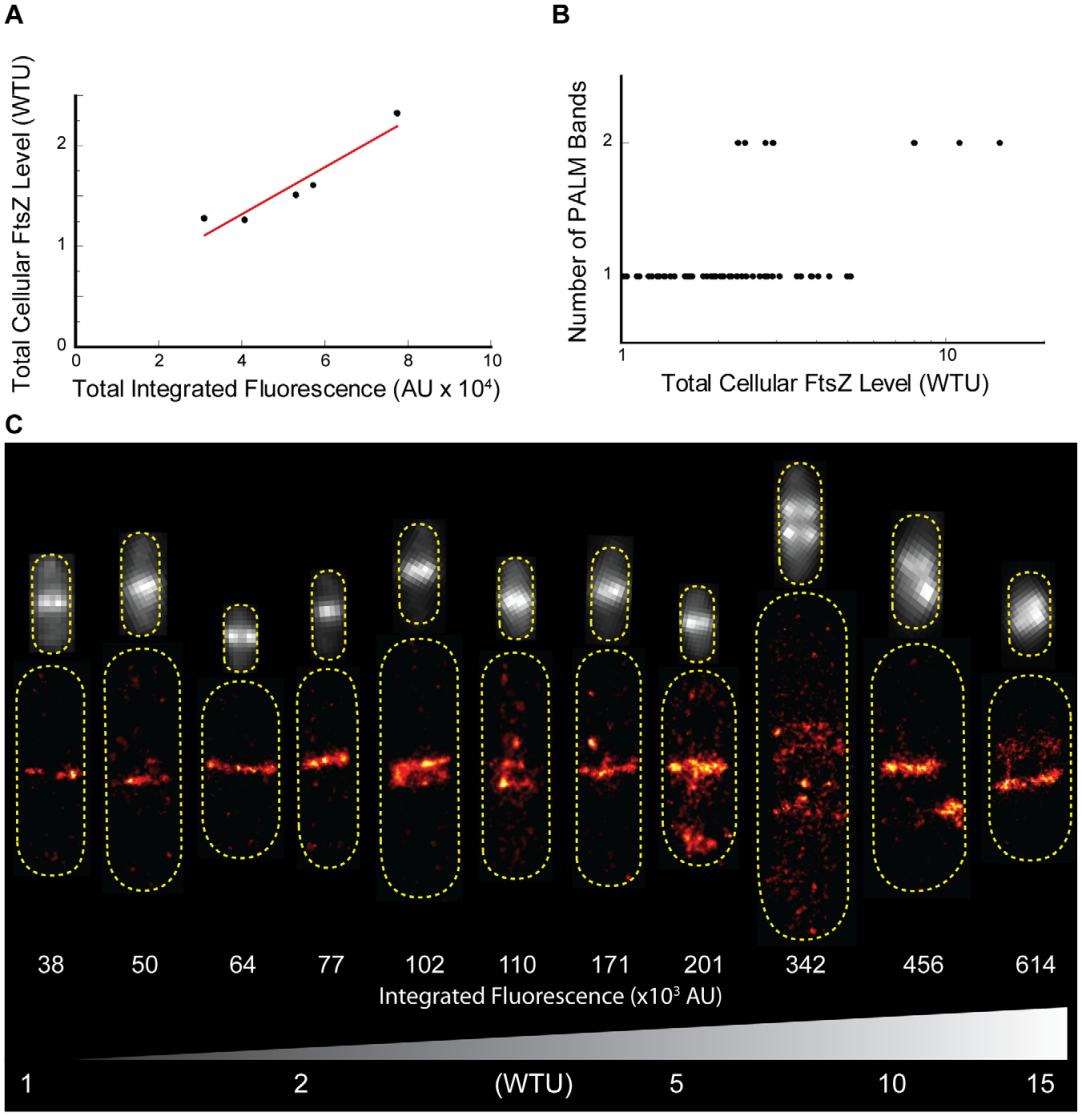
Dependence of Z-ring conformation on FtsZ expression level. A. Correlation plot showing the linear relationship between the total FtsZ expression level (wt FtsZ + FtsZ-mEos2) of pulse-induced B/r A/pCA24N cells determined by immunoblotting and the corresponding average integrated fluorescence intensity. A linear least squares fit was applied and this conversion was used to estimate the total cellular FtsZ expression level from the integrated ensemble fluorescence (y = 0.4+2.32×10^−5^×, R^2^ = 0.898). B. Plot of Z-ring conformations observed in the PALM images, represented as number of bands, versus total cellular FtsZ level in WTU (log-scale), illustrating that most cells with low-to-intermediate expression levels exhibit single bands, while high expression levels result in exclusively multiple-band conformations. C. Representative PALM plots (bottom) and ensemble fluorescence images (top) are labeled with and arranged according to increasing total integrated fluorescence (AU×10^3^) to illustrate the dependence of Z-ring conformation on FtsZ expression level. A gradient corresponding to the increase in total FtsZ expression level (WTU) is displayed at the bottom. Ensemble fluorescence images are not displayed according to scale and are presented according to the contrast defined by the dynamic range of the individual cell.

We first established a standard curve so that the total cellular FtsZ level of an individual cell can be easily inferred from its integrated green fluorescence of FtsZ-mEos2 instead of relying on an average expression level measured on a large population of cells using immunoblotting (Figure 6A). To obtain a wide range of total cellular FtsZ levels, we used the B/r A strain which harbors the *ftsZ-mEos2* fusion gene under the control of the *lac* promoter on a multicopy plasmid pCA24N and employed a “serial dilution by division” strategy. We pulse-induced the expression of FtsZ-mEos2 and then washed off the inducer. The cells were allowed to continue growing so that the induced FtsZ-mEos2 levels naturally decreased each time the cell divided. At different time points after induction was halted, we harvested cells and quantified both the average expression level of FtsZ-mEos2 using immunoblotting and the corresponding average integrated fluorescence of a population of single cells using fluorescence microscopy. Assuming a constant expression level of wt FtsZ from its chromosomal locus, we plotted total cellular FtsZ level (in units of wt FtsZ level, WTU) against the average integrated green fluorescence of the cells (Figure 6A). This standard curve allowed us to analyze the structural properties of the Z-ring in accordance with the total cellular FtsZ level in individual cells. We note here that in order to use fluorescence as an expression marker we varied the total cellular FtsZ level by changing the levels of FtsZ-mEos2 instead of wt FtsZ. Therefore, the structural changes we observe in following experiments could be due to the mEos2 tag instead of FtsZ. Although this remains a formal possibility, previously it has been shown that overexpression of FtsZ or FtsZ-FP fusion proteins led to similar spiral Z-ring conformations [8], [13], suggesting that the structure of the Z-ring is mainly influenced by FtsZ, not the FP tag.

To analyze the expression level dependence of Z-ring conformation, we plotted the number of PALM bands observed against the total integrated green fluorescence intensity for all B/r A cells (Figure 6B). As with the BL21(DE3)pLysS strain, we observed two types of cells exhibiting either single or double bands at the midcell in the B/r A strain. In Figure 6C, PALM images of a few representative cells are arranged according to their integrated green fluorescence intensity recorded before PALM imaging. In the intensity range of 25,000–70,000, which corresponds to a total cellular FtsZ level of 1–2 WTU, all cells (N = 29) exhibited a single band, presumably a ring-like conformation, at the midcell. In the intensity range of 70,000–200,000, which corresponds to a total cellular FtsZ level of 2–5 WTU, a few cells (4 out of 26) showed more than one PALM band at the midcell, reminiscent of the compressed helical structures observed in the BL21(DE3)pLysS strain. The ensemble green fluorescence images of the cells exhibiting multiple PALM bands still showed one single band. When the fluorescence intensity is above 300,000 (total cellular FtsZ level >8 WTU, N = 3), we found that both the ensemble green fluorescence images and PALM images of cells showed multiple, widely-spaced bands at the midcell.

Taken together, the above observations suggest that the Z-ring has an increased tendency to form helical structures at elevated expression levels of FtsZ. However, at the intermediate expression level (2–5 WTU) the Z-ring may constantly transition between the ring-like and compressed helical conformations, whereas at high expression level (>8 WTU) the Z-ring predominately exists as helical structures that are visible to conventional FLM, similar to previous observations when FtsZ was highly overexpressed [8], [13]. It was shown that a two to seven-fold increase in FtsZ expression level led to enhanced cell division exemplified by the formation of mini cells [42] and that cell division became inhibited when FtsZ expression was increased to more than 10-fold the endogenous level [13], [42]. Based on these studies, we reason that it is likely that the compressed helical conformations we observed at the intermediate concentration range do not inhibit the constriction function of the Z-ring, Instead, they reflect the dynamic nature of the Z-ring because some cells that have even higher expression levels of FtsZ still showed a single ring-like conformation at the midcell (Figure 6B–C). Indeed, by conventional time-lapse FLM experiments we found that even after the Z-ring has stably assembled at the midcell, the Z-ring is still remarkably dynamic—the midcell band sometimes split in two, was oriented at an angle or appeared helical despite limited spatial resolution (Figure S4). Cells showing these dynamic helical conformations divided without detectable defect. Further experiments examining the dynamics of the helix-ring transition with subdiffraction-limited spatial resolution will provide important insight into the structural organization of the Z-ring.

**Figure 7:**
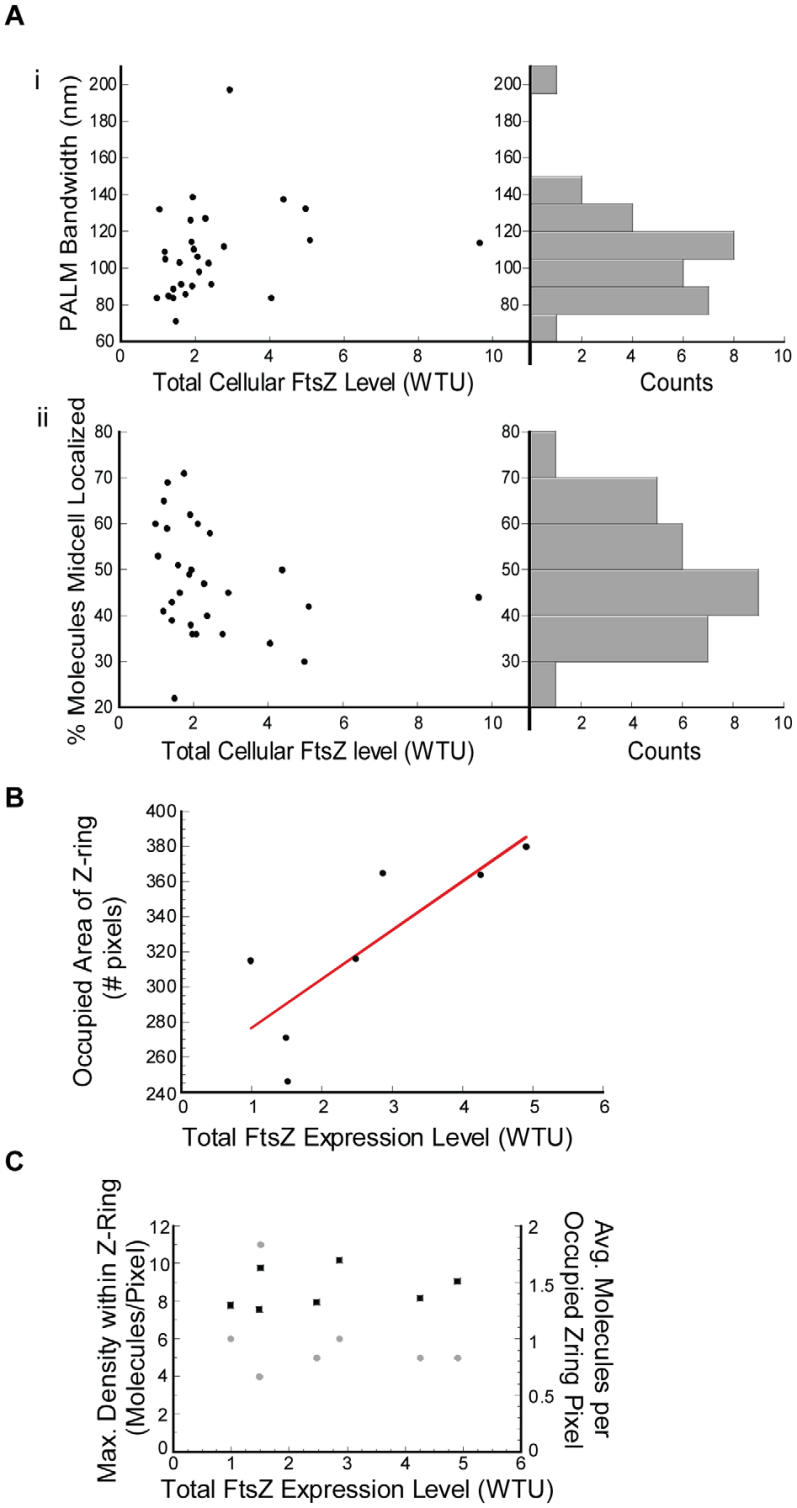
Dependence of Z-ring properties on FtsZ expression level. A. PALM bandwidth (i) and the percent of molecules localized to the Z-ring (ii) are plotted against the total cellular FtsZ expression level in WTU, indicating that higher expression of FtsZ leads to an increased number of FtsZ molecules in the Z-ring, but not to a wider Z-ring. Each plot is conjoined on the right with a corresponding histogram. The width of individual PALM bands was measured in B/r A cells according to the procedure outlined in Figure S3. The percent of molecules localized to the Z-ring was measured in B/r A cells via a custom MatLab (MathWorks) routine that required the Z-ring to be manually outlined by the user. B. Correlation plot showing the linear relationship between the total pixel area of the Z-ring, calculated from the PALM image as the number of pixels containing molecules within the user-defined region, and the total FtsZ expression level. This plot indicates that additional FtsZ molecules preferentially fill in empty spaces in the Z-ring. C. Maximum (gray circles) and average (black squares) Z-ring density are plotted against total FtsZ expression level. Maximum Z-ring density refers to the highest number of detected FtsZ-mEos2 molecules in a single pixel within the user-defined Z-ring boundary. Average Z-ring density is calculated by dividing the total number of FtsZ-mEos2 molecules detected in the Z-ring by the number of pixels they occupied.

Next, we analyzed how the width of the Z-ring responds to elevated FtsZ levels. In contrast to the conformation of the Z-ring, we found that the width of the Z-ring does not exhibit clear concentration dependence. In Figure 7Ai we plotted the measured PALM band width against the FtsZ expression level of each cell. No clear trend can be observed. The average bandwidth of all B/r A cells is 108±25 nm (N = 29). Since the Z-ring bandwidth of B/r A cells expressing different levels of FtsZ-mEos2 is not significantly different from what was measured on the BL21(DE3)pLysS cells described above (p = 0.48), we combined all the data together and the resulting histogram of band width distribution is shown in Figure 7Ai. The average band width is 110±25 nm for both strains.

The observation of a constant width of the Z-ring irrespective of the total cellular FtsZ level could be due to two different possibilities. The first possibility is that only a fixed number of FtsZ molecules are allowed to localize to the midcell to form a Z-ring regardless of the total cellular FtsZ expressed. The second possibility is that the Z-ring is a loose structure and it naturally occupies a region of ∼110 nm at the midcell. When the total cellular FtsZ level increases, extra FtsZ protofilaments either fill in the empty spaces of the loose structure or aggregate onto existing protofilaments of the Z-ring.

To examine the first possibility, we calculated the percentage of total cellular FtsZ localized to the midcell of B/r A cells by dividing the number of FtsZ-mEos2 molecules detected in the Z-ring by the total number of molecules detected in the whole cell. If only a fixed number of FtsZ molecules are allowed at the midcell, at increased expression levels of FtsZ we would observe a decreased percentage. However, we found that the percentage is largely invariable across the concentration range we examined (Figure 7Aii). The average percentage, (47±12)%, agrees within experimental error with previous measurements based on conventional FLM (30–40%) [16], [43]. The invariable percentage of FtsZ molecules localizing to the Z-ring also shows that increased FtsZ expression levels result in more FtsZ molecules localized to the midcell. Therefore, the constant width of the Z-ring is not due to a fixed number of FtsZ molecules at the midcell.

Next, we examined the second possibility by measuring the density of the Z-ring of B/r A cells expressing different levels of FtsZ using TIRF-PALM imaging. We first recorded the ensemble green fluorescence of each cell using epi-illumination mode. We then switched to TIR illumination to record the corresponding PALM image. As expected, we found that the width of the Z-ring measured using TIRF is independent of total cellular FtsZ expression level with an average of 108±17 nm (N = 7). However, the area of the Z-ring that is occupied by FtsZ-mEos2 molecules, showed a linear dependence on the total cellular level of FtsZ (Figure 7B). Interestingly, within these occupied areas the average and maximal numbers of FtsZ-mEos2 molecules detected per PALM pixel are largely constant (Figure 7C). These results strongly suggest that at increased FtsZ expression levels the Z-ring becomes denser because additional FtsZ molecules occupy otherwise empty space inside the Z-ring instead of aggregating onto existing FtsZ protofilaments. In other words, the Z-ring at the normal wt FtsZ expression level is most likely a loose 3D structure.

## Discussion

### The helical and ring-like conformations of the Z-ring

The midcell Z-ring has long been viewed as a closed circle. Here, using PALM, we imaged the Z-ring of *E. coli* with ∼35-nm spatial resolution. We discovered that in addition to the ring-like conformation, the Z-ring of *E. coli* may adopt a variety of compressed helical conformations with tight pitches and variable lengths. These compressed helical structures have a higher tendency to form at elevated FtsZ expression levels (2–5 WTU). When viewed under a conventional fluorescence microscope, these helical conformations are indistinguishable from the ring-like conformation. When the expression level of FtsZ exceeded ∼8-fold of WTU, however, helical structures with larger pitches were observed even under conventional FLM. We believe these structures are reminiscent of the previously reported extended helical structures when FtsZ is highly overexpressed [13].

We note that the compressed FtsZ helical structures we observed have smaller pitches, shorter lengths and a different midcell localization pattern than the extended FtsZ helical structures previously reported [10]–[12]. Nevertheless, it was suggested that the extended helical structures may condense to the midcell, hinting that these more extended helical structures may be related to the midcell helical structures we observed [10]–[12]. We were unable to verify this hypothesis because we did not observe extended FtsZ helical structures in the PALM images of cells with low to intermediate FtsZ expression levels. The absence of extended FtsZ helical structures in these cells could be due to our limited sample size, but most likely it is due to the transient, low-density nature of these structures in cells containing only a moderate excess (2–3 fold WTU) of FtsZ [12].

### Assembly mechanism of variable FtsZ helical structures

We are interested in understanding why FtsZ assembles into variable helical structures at the midcell. One possibility is that there may be an existing helical pathway on the membrane upon which FtsZ assembles. This possibility has been suggested for extended FtsZ helical structures formed outside of the midcell region [10]–[12], [44] and other cytoskeletal proteins such as MinD and MreB [45]–[48]. Supporting this possibility is the discovery of lipid spirals on the membrane of *B. subtilis*, and that MinD was found to colocalize with these lipid spirals [45]. However, these lipid spirals were not observed in *E. coli* and they were static on the time scale of tens of minutes, inconsistent with the dynamic nature of the Z-ring [45]. A second possibility is that a helix is the natural result of a polymer growing on the cylindrical surface of a rod-shaped cell. The shape of this helix is governed only by the polymer's intrinsic mechanical properties and interactions with surrounding proteins and membrane lipids, which, if not uniform along the helical path, give rise to variable pitch and length of the helix [44]. This model also explains the formation of a ring, which is the direct result of the polymer growing perpendicularly to the long axis of the cell.

We favor the second model because the midcell FtsZ helical structures we observed had variable pitches and lengths, which could have potentially resulted from interactions of FtsZ with the surrounding cellular environment such as proteins and lipids. Several pieces of evidence support this hypothesis. First, FtsZ assembles into protofilaments and higher-order structures *in vitro* with a wide range of shapes, curvatures and geometries under different experimental conditions. This polymorphism indicates that the assembly of FtsZ structures is sensitive to its surrounding environment. Second, it was observed that the FtsZ monomer itself and the interface of subunits in protofilaments are flexible [49]. Hence, polymerized FtsZ may adopt different bending angles according to its local environment. Third, several proteins, including MinC, SulA, FtsA, ZipA, SlmA, ZapA and ZapB, modulate FtsZ assembly and disassembly by promoting or disrupting FtsZ polymerization [50]–[54]. These modulations may occur at various stages during the cell cycle or at various positions along the helical path, leading to an FtsZ helix with variable lengths and pitches. Finally, more than ten division proteins, including the aforementioned FtsA and ZipA, are believed to localize to the Z-ring as small clusters [55]. The non-uniform presence of these division proteins may also change the Z-ring's mechanical properties so that the interactions of FtsZ with the membrane are not uniform, potentially resulting in various helical structures [44].

### Arrangement of protofilaments inside the Z-ring

We were unable to resolve individual protofilaments inside the Z-ring, which is likely due to our limited spatial resolution (∼35 nm). We note that the spatial resolution we reported here is the 2D resolution as PALM images are 2D projections of 3D structures. Therefore, if protofilaments are arranged side-by-side in a single layer parallel to the imaging plane, and the separation between protofilaments is less than 35 nm, we will be unable to distinguish individual protofilaments. However, if protofilaments overlap with each other along the radial direction of the cell, the projected separation between protofilaments would be less than the 2D spatial resolution, preventing the resolution of single protofilaments.

**Figure 8:**
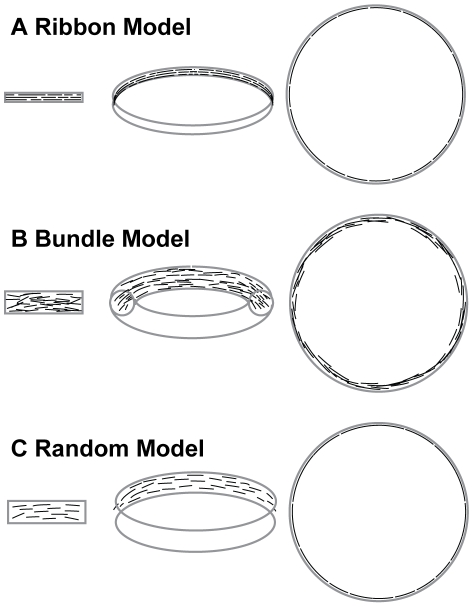
Schematic drawings of arrangement of protofilaments inside the Z-ring. A. Flat-ribbon model. B. Loose bundle model. C. Random model. In each model, the 3D view of the arrangement of FtsZ protofilaments is shown in the middle. The 2D projection of a section of the ring along the long axis of the cell is shown on the left. The cross section of the ring along the short axis of the cell is shown on the right. FtsZ protofilaments are shown as black short arcs and the overall boundary of the Z-ring is outlined in gray. In the flat-ribbon model, FtsZ protofilaments are arranged side-by-side in a single layer. They may or may not be connected head-to-tail to make longer filaments. In the loose bundle model, FtsZ protofilaments loosely associate with each other via non-uniform lateral interactions mediated by the intrinsic affinity between protofilaments and/or bundle-promoting proteins such as ZipA, ZapA and ZapB (not pictured). Individual FtsZ protofilaments are not necessarily aligned with each other and they may have different curvatures. In the random model, FtsZ protofilaments randomly scatter in a narrow band at the midcell and there are large spaces separating individual protofilaments, which do not engage in lateral interactions. Note that the sizes of the cell, the Z-ring and FtsZ protofilaments are not drawn to scale in order to enlarge details in the arrangement of FtsZ protofilaments.

Although we could not resolve individual protofilaments inside the Z-ring, our measurements provide insight into the arrangement of protofilaments. Based on the average number of FtsZ molecules per cell determined by immunoblotting (4100 molecules/cell) and the percentage of FtsZ localized to the midcell (47%), we estimated that the wt midcell Z-ring of the *E. coli* BL21 and B/r A strains would contain ∼1900 FtsZ molecules. If all these FtsZ molecules were connected head-to-tail, they would form a filament long enough to encircle the waist of a typical *E. coli* cell 2–3 times. Therefore, the Z-ring is ∼2–3 protofilament thick [15]. If these protofilaments were arranged side-by-side in a flat ribbon configuration with a 9-nm spacing between each protofilaments [22], the width of the Z-ring would be 20–30 nm. This number is in contrast to what we measured using PALM imaging (∼110 nm). It is more likely that these protofilaments are not aligned tightly but loosely arranged inside the Z-ring as illustrated in Figure 8B. Because of the relatively short length of FtsZ protofilaments (∼120 nm on average [15]–[17]) compared to that of the curvature of the cell membrane, it is also possible that FtsZ protofilaments are oriented in all directions at the midcell, not necessarily only along the waist of the cell. The random orientations of FtsZ protofilaments would also increase the apparent width of the Z-ring measured by PALM imaging. Interestingly, although the width measurements indicate that the Z-ring is most likely a loose structure, our TIRF PALM imaging showed that in some areas of the Z-ring, FtsZ protofilaments have higher packing density than what is expected for a single-layered configuration. This observation indicates that the Z-ring may not be a homogenous structure but in some areas FtsZ protofilaments overlap with each other in space. In addition, the concentration-dependent PALM imaging experiment showed that when additional FtsZ protofilaments are added to the Z-ring, they do not increase the apparent width of the Z-ring but fill in between existing protofilaments, further supporting the hypothesis that the Z-ring is a loose structure.

Considering the above measurements and observations, we propose that the most likely arrangement of protofilaments inside the Z-ring is a loose 3D bundle in which randomly oriented FtsZ protofilaments loosely associate and overlap with each other in space (Figure 8B). The bundle allows FtsZ protofilaments to overlap with each other so that when the structure is projected onto a 2D plane the FtsZ packing density is higher than a single-layered ribbon configuration. The bundle is also loose so that its projected width (110 nm) is larger than that of a structure in which the same number of protofilaments are aligned side-by-side as illustrated in the ribbon model of Figure 8A. The ribbon model also predicts that at increased expression levels of FtsZ we would observe increased width but not density of the Z-ring since additional FtsZ protofilaments can only be added to the two edges of the ribbon. This prediction is the opposite of what we observed.

A previous model proposed that the Z-ring is comprised of short protofilaments randomly scattered in a narrow band around midcell (Figure 8C) [22], [29]. In this model there are large spaces between FtsZ protofilaments along the membrane and hence there are no lateral associations [29]. The loose bundle model we propose is similar to this model in that we believe the Z-ring is also a loose structure with randomly oriented FtsZ protofilaments. However, our model is different in that we speculate that short FtsZ protofilaments inside the Z-ring are staggered with each other in space—not only along the circumference of the cell, but also along the radial direction of the cell (Figure 8B). This model brings forth an interesting prediction—lateral interactions among protofilaments are required to organize such a 3D bundle, since protofilaments facing the cytoplasmic side may not be anchored to the membrane.

Currently the presence of lateral interactions between FtsZ protofilaments inside the Z-ring remains controversial. Lateral surfaces of FtsZ are different from those of its homolog tubulin, which exhibits strong lateral interactions [56], and the lateral bond energy of FtsZ was estimated at only about 1/50 of that of the longitudinal bond [17], [57], [58]. However, a few results argue strongly for the presence of lateral interactions between FtsZ protofilaments. First, an FtsZ mutant that is defective in making lateral contacts *in vitro* does not support cell division [59]. Second, the MinC protein, a known cell division inhibitor, was found to prevent lateral interactions between FtsZ filaments *in vitro* [60]. Finally, two newly discovered cytoplasmic proteins, ZapA and ZapB, are found to interact with FtsZ and promote Z-ring assembly *in vivo* [51], [54], [61]–[63]. These proteins may serve as the “glue” that mediates the lateral associations of FtsZ protofilaments.

If there are indeed lateral associations between FtsZ protofilaments, we reason that they are likely weak and not uniformly distributed along the longitudinal directions of protofilaments. The weak lateral associations between FtsZ protofilaments are necessary for a loose bundle and may facilitate the dynamic structural remodeling of the Z-ring between the helical and ring conformations. The non-uniform lateral associations may arise from varying curvatures of protofilaments caused by the mixed contents of GTP and GDP in protofilaments [20]. Slightly curved FtsZ protofilaments have been observed *in vitro* regardless of bound nucleotide states [6], [20], [31], [57], [64], [65]. Loosely associated FtsZ higher-order structures including bundles, helices, toroids and rings formed by lateral interactions in the presence of molecular crowding agents have also been observed *in vitro* [5]. In addition, a recent structural study showed that FtsZ monomers and protofilament interfaces are flexible, which allows FtsZ protofilaments to adopt different curvatures by bending at the interfaces or within the subunits themselves [49].

In summary, our PALM images of the Z-ring point to a loose 3D bundle model in which FtsZ protofilaments weakly associate with each other via non-uniform lateral interactions. The presence of lateral interactions between FtsZ protofilaments allows the formation of organized, multi-stranded Z-ring conformations such as helices in the absence of a pre-existing track on the cell membrane. The looseness of the bundle may also help to explain why the Z-ring of *E. coli* is invisible using EM or ECT, which usually detects densely- or regularly-packed cellular structures. To validate this model, further experiments investigating the influence of lateral interactions between FtsZ protofilaments on the structural organization of the Z-ring are required.

## Materials and Methods

### Strains and plasmids

*E. coli* strain BL21(DE3)pLysS was from Stratagene (Stratagene, LA, Jolla, CA) and used for PALM imaging. Strain JKD7-2/pKD3a for the complementation assay was kindly provided by Dr. J. Lutkenhaus, University of Kansas Medical Center. To construct the pET21b-mEos2 plasmid, mEos2 was amplified from pRSET-mEos2 plasmid (gift from McKinney [26]) using primers GATCGCTAGCATGAGTGCGATTAAG and GATCCTCGAGTTATCGTCTGGCATTGTC, restricted with *Nhe*I and *Xho*I (underlined), and ligated into a similarly digested pET21b plasmid (Novagen). To construct the pET28-FtsZ-mEos2 plasmid, we first produced the pET28-FtsZ-rsFastLime plasmid using the pET28-M13-rsFastLime plasmid (gift from Egner)[66] by swapping the coding sequence of MCP with that of FtsZ. The coding sequence of FtsZ was amplified from plasmid pCA24N-FtsZ (NBRP (NIG, Japan):*E. coli*) using primers ATGTCATATGTTTGAACCAATGGAACTTACC and ATTAGGATCCATCAGCTTGCTTACGCAG, digested with *Nde*I and *Bam*HI (underlined), and ligated into the similarly digested pET28-M13-rsFastLime plasmid. FtsZ was then cut from pET28-FtsZ-rsFastLime using restriction enzymes *Xba*I and *Bam*HI. The product was ligated into a similarly digested pET28-M13-mEos2 plasmid, which was constructed by swapping the coding sequence of rsFastlime with mEos2. Specifically, mEos2 was amplified using primers GATCGAATTCATGAGTGCGATTAAG GATCCTCGAGTTATCGTCTGGCATTGTC, restricted with *Eco*RI and *Xho*I (underlined), and ligated into a similarly digested pET28-M13-rsFastLime plasmid to replace the rsFastLime fragment. The linker sequence between FtsZ and mEos2 is GSAGSAAGSGEF [66]. The constructed plasmids pET21b-mEos2 and pET28-FtsZ-mEos2 were then transformed into the BL21(DE3)pLysS strain.

### Growth conditions

Cells harboring the pET21b-mEos2 plasmid were cultured at 37°C in Luria-Bertani (LB) medium overnight in a shaker (250 rpm). Cells were then diluted (1∶1000) in M9 minimal media supplemented with 0.4% glucose, MEM vitamins and amino acids (Sigma) and cultured at room temperature (RT) with shaking. When the OD_600_ reached ∼0.3, the cell culture was induced with 20 µM Isopropyl β-D-1-thiogalactopyranoside (IPTG) for 16 hr. Cells harboring pET28-FtsZ-mEos2 were cultured in LB overnight at RT, and reinoculated in supplemented M9 media the next morning. When the culture's OD_600_ reached ∼0.4, 50 µM IPTG was added for 30 min. After induction, cell cultures were harvested by centrifugation and pellets were washed twice with the M9 medium. Cells were then fixed with 4% formaldehyde (1 volume of growth medium +1 volume of 8% formaldehyde) for 40 min at RT. After fixation, cells were washed twice with M9 medium before PALM imaging.

### Plate complementation assay

A plasmid expressing FtsZ-mEos2 was transformed into JKD7-2/pKD3a [67] to examine if FtsZ-mEos2 is able to complement the depletion of wild-type (wt) FtsZ expressed from the temperature sensitive plasmid pKD3a at 42°C. The chromosomal copy of *ftsZ* was disrupted by the insertion of a kanamycin gene in JKD7-2 strain. To remove the expression dependence of T7 RNA polymerase of the pET28-FtsZ-mEos2 plasmid, the *ftsZ-mEos2* fragment was amplified by PCR from the pET28-FtsZ-mEos2 plasmid using primers CTATGGCCCTGAGGGCCATGTTTGAACCAATGGAACTTACC and AATTGCGGCCGCTTATCGTCTGGCATTGTCAG. The amplified fragment was gel-purified (Qiagen), digested with *Sfi*I *and Not*I (underlined) and subcloned into the same sites of pCA24N (NBRP (NIG, Japan):*E. coli*), generating pJB004. Clones were confirmed via sequencing and the plasmid pJB004 was transformed into JKD7-2/pKD3a cells.

Plate complementation assays were performed on JKD7-2/pKD3a/pJB004 cells grown to mid-log phase in LB media containing a range of IPTG concentrations (0–1.0 mM). Serial dilutions of the cultures were then plated on corresponding IPTG plates in duplicate and grown at permissive (30°C) and nonpermissive (42°C) temperatures. Numbers of colonies grown at 42°C and 30°C at each IPTG concentration were then compared. At all IPTG concentrations tested no growth was detected at 42°C, indicating that FtsZ-mEos2 did not complement the conditional depletion of wt FtsZ. We have also constructed a series of FtsZ-mEos2 fusion proteins by either changing the linker sequences between FtsZ and mEos2 or by placing mEos2 at the N-terminus or internal positions of FtsZ. However, none of these fusion proteins complemented the JKD7-2/pKD3a strain at nonpermissive temperature.

### Quantification of endogenous FtsZ expression level

To obtain a standard for the quantification of the endogenous FtsZ expression level in the BL21(DE3)pLysS and the B/r A strains, we constructed an FtsZ purification vector that allowed for His-tag facilitated affinity purification. The *ftsZ* gene was amplified from pJB004 using primers AATTGTCGACAATGTTTGAACCAATGGAACTTAC and TTAAGCGGCCGCTTAATCAGCTTGCTTACGCAG, was restricted with *Sal*I and *Not*I (underlined), and subcloned into the same sites of pT7HMT [68] creating pJB041. The plasmid was confirmed via sequencing and transformed into BL21-Gold(DE3) (Stratagene). To purify FtsZ, cells were cultured overnight at 37°C in LB media. The culture was then diluted 1∶500 in 150 mL LB media, grown at 37°C to an OD_600_ of 1.0, then induced for 2 hours with 0.5 mM IPTG. Cells were collected at 4100 rpm for 15 min in a Thermo Legend bench top centrifuge at 4°C. Cells were then processed according to the ProBond™ native protocol (Invitrogen). After elution, the pooled sample was dialyzed in 1× ProBond Native Buffer and simultaneously incubated with 300 ug of TEV Protease (a gift from Dr. Leahy, Johns Hopkins University). Cleavage of the 6xHis-Myc-TEV tag was confirmed via SDS-PAGE electrophoresis and the sample was reapplied to native ProBond to remove the His-tagged TEV Protease. Pooled samples were dialyzed against FtsZ Storage Buffer (20 mM Tris-HCl, 250 mM KCl, 1 mM EDTA, 10% Glycerol) and stored at −80°C. Purified FtsZ was determined to be ∼95% pure via Coomassie staining. A BCA assay (Pierce) was performed to determine the concentration of the purified FtsZ.

BL21(DE3)pLysS and B/r A cells were grown in supplemented M9 media at RT to mid-log phase. Cells were processed for immunoblotting as described above, except that prior to snap-freezing cell counting was performed using a Petroff-Hausser counting chamber via an Olympus CKX41 microscope equipped with an LCAch N 20× objective and WHB10× eyepiece. Immunoblotting was performed in duplicate as described above with the addition of a set of FtsZ standards. A quantitative comparison was performed using a Typhoon imager (Figure 1A). From our results we conclude that BL21 and B/r A are similar in their respective FtsZ expression levels and that on average, the expression level of wt FtsZ under the growth conditions used for PALM imaging is 4,100±1,700 molecules/cell.

### Expression level of FtsZ-mEos2 in BL21(DE3) pLysS strain

To assess the expression level of FtsZ-mEos2 under our PALM imaging condition, we determined the ratio of expressed FtsZ-mEos2 to total cellular FtsZ level (wt FtsZ + FtsZ-mEos2) for BL21(DE3)pLysS cells harboring the pET28-FtsZ-mEos2 plasmid. Cells were grown according to the growth and induction conditions used for PALM. After induction, samples were centrifuged, resuspended in 100 µl of supplemented M9 medium, and snap frozen. Thawed samples were boiled in 300 µl of Western Sample Buffer (1 ml 1× Laemmli Buffer, 700 µl 3× Laemmli loading dye, 300 µl 20% (w/v) SDS, 70 µl 1 M DTT), separated on 10% SDS-PAGE gels (Bio-Rad), semi-dry transferred to Trans-blot nitrocellulose membranes (Bio-Rad) and blocked overnight in 5% Non-fat Milk/TTBS (20 mM Tris pH 7.5, 150 mM, NaCl, 0.1% Tween). Primary incubation was with a affinity purified anti-FtsZ rabbit antibody (gift from Dr. Erickson, Duke University) diluted 1∶1500 in TTBS +1% BSA for 120 min. Secondary incubation was with Goat anti-Rabbit HRP Conjugate (Bio-Rad) diluted 1∶45,000 in TTBS for 60 min. Blots were treated with the Immun-Star WesternC™ chemiluminescence kit (Bio-Rad) and signal was detected either by autoradiography film or a Typhoon 9410 variable mode imager. Autoradiography films were subsequently scanned using a Canon Scanner (CanoScan LiDE 30). Image analysis was performed using ImageJ (NIH). Blots were performed in duplicate. We found that on average (six repeats) FtsZ-mEos2 was expressed at (25±7) % of total cellular FtsZ (Figure 1A).

In addition to the average expression level of FtsZ-mEos2 in the BL21(DE3)pLysS strain, we also measured the distribution of the expression level of FtsZ-mEos2 in individual BL21(DE3)pLysS cells by integrating the total green fluorescence of FtsZ-mEos2 in individual cells. The average fluorescence intensity (arbitrary unit) of the whole population is 21800±9700 (N = 129, CV = 0.44). The average fluorescence intensity of the subpopulation of cells showed midcell FtsZ localization is 26000±11000 (N = 13, CV = 0.43), not significantly different from that of the whole population. On average there are only ∼10% of cells showing distinct midcell FtsZ localization. The low percentage of cells showing midcell FtsZ localization compared to that of the B/r A strain (∼80%) could be due to the difference in the localization time of FtsZ during the cell cycle and/or the overall expression levels of FtsZ-mEos2 in the two strains. For example, in a different strain (AG1) we found that the midcell localization percentage of FtsZ is near 100% because we observed the formation of new Z-rings immediately following cell division.

### Correlating FtsZ-mEos2 expression with integrated green-channel fluorescence

B/r A cells containing pJB004 were grown in supplemented M9 media at 25°C. The overnight culture was diluted 1∶500 in 50 mL of supplemented M9 at 25°C and grown to an OD_600_ of 0.2, at which time the culture was induced with 15 µM IPTG for 2 hrs. Cells were then collected via centrifugation and resuspended with fresh supplemented M9 media. This process was repeated one more time to remove the inducer. The cells were then returned to growth at RT and processed at 30-min intervals for a total of 4 hrs for immunoblotting, live-cell fluorescence imaging and fixed cell (4% formaldehyde at RT for 40 min) PALM imaging. The culture was diluted as needed to maintain steady-state growth.

Western samples were processed as described above. The concentration of FtsZ-mEos2 was determined relative to that of wt FtsZ. Single-cell fluorescence imaging was performed as described previously [69]. A large sample size of live cells (N>100) were analyzed at each time point via ensemble (green channel) epi-fluorescence. Assuming the endogenous wt FtsZ expression level is independent of the expressed FtsZ-mEos2 level, the relative expression level of FtsZ-mEos2 over wt FtsZ level determined by immunoblotting could then be converted to the total cellular FtsZ level (FtsZ-mEos2+ FtsZ) and correlated with the average fluorescence intensity level of the same cell population at each time point.

### Comparison between FtsZ-mEos2 and FtsZ-GFP fusions

To assess if FtsZ-mEos2 behaves similar to FtsZ-GFP, we transformed plasmids pJB004 and pCA24N-FtsZ-GFP [70] into *E. coli* strain B/r A. These two cell strains and the parent strain B/r A were prepared for conventional fluorescence time-lapse imaging as described previously [69]. Time-lapse imaging was performed on cells sandwiched between a coverslip and an agarose gel pad made with M9 growth media at room temperature (RT) (Figure 1C). The microscope (Olympus IX81) was equipped with a PlanApo 100× (1.45 NA) objective, an Andor iXon model 888 CCD camera and a standard GFP filter set from Chroma. Bright-field and fluorescence (green channel) images of cells were acquired every 2 min using MetaMorph (Molecular Devices). The time-lapse movies of the FtsZ-mEos2- and FtsZ-GFP-expressing strains showed essentially the same cell-cycle dependent localization patterns of FtsZ at the midcell (Figure 1C). Average cell doubling time for the FtsZ-mEos2-, FtsZ-GFP-expressing strains and the parent strain were determined from the corresponding time-lapse movies (Figure 1D). Average cell lengths (Figure 1D) and the percentages of cells showing midcell FtsZ localization pattern were determined from snap shots of cells in steady-state.

### PALM imaging

Fixed cells were mixed with 50 nm diameter gold beads (Microspheres-Nanospheres, Mahopac, NY), pipetted between two coverslips, and allowed to settle for 30 min at RT in an assembled observation chamber (FCS2, Bioptechs, Butler, PA) prior to PALM imaging. The chamber was then locked on the microscope stage (ASI, Eugene, OR) and maintained at RT throughout imaging. Images were acquired using an Olympus IX-71 inverted microscope, equipped with a 60×, 1.45 NA TIRFM objective. A 405-nm laser (CUBE™, Coherent, Santa Clara, CA) was used to activate the fluorescent protein mEos2. The green fluorescence of mEos2 was excited with an argon-ion laser at 488 nm (Innova 308C, Coherent, Santa Clara, CA) and emission was collected with a dual-band emission filter (510/19 and 620/20, Chroma Technology, Rockingham, VT). The red fluorescence of mEos2 was excited with a tunable dye laser at 570 nm (Model 599, Coherent, Santa Clara, CA) with an excitation density of 700 W/cm^2^. Emission was collected with the same dual-band emission filter. Both activation and excitation beams illuminated the sample in epi-illumination mode unless otherwise noted. The sample was illuminated continuously with 570-nm laser and the integration time was 30 ms for each frame. This illumination method provided imaging speed of 33 frames s^-1^ for an image size of 100×100 pixels. As the number of inactive mEos2 molecules decreased during data acquisition, the intensity of the activation laser was increased stepwise. Images were acquired with an EMCCD camera (IXON DU897E, Andor Technology, Belfast, Northern Ireland). The 50-nm luminescent gold beads were used to calibrate for stage drift during data acquisition. Individual fluorescent spots of the beads were fitted and localized in the same manner as single molecules of mEos2 [24]. Objective-type TIR PALM imaging was performed by translating the laser beams away from the central axis of the objective using a set of mirrors in the optical pathway. Construction of PALM images was carried out according to what was described previously (Text S1) [24].

## Supporting Information

Text S1 Construction and spatial resolution of PALM images.
(0.02 MB DOCX)

Text S2 Modeling of 3D helices and simulations of PALM images.
(0.02 MB DOCX)

Figure S1 Localization precision and spatial resolution of PALM images. A. Histogram of all the localization precisions for all the molecules detected from both the B/r A and BL21(DE3)pLysS fixed samples (mean = 17 nm), As indicated by the red line, only molecules possessing a localization precision of 25 nm or better were used to construct fixed PALM images. B. Histogram of total number of photons detected for all the FtsZ-mEos2 molecules detected in fixed B/r A and BL21 samples (mean = 910 photons). C. Displacement distribution of single FtsZ-mEos2 molecules that lasted more than one frame in PALM imaging sequences. The positions generated by the same molecule in consecutive frames reflect the error in the position determination of single molecules, i.e., the actual spatial resolution of PALM imaging. The distribution of the displacement was fit with the sum of two normal distributions with the major peak at 33.9 nm.
(0.23 MB TIF)

Figure S2 The helical model used to simulate the PALM images in Figure 3. A. A helix (red) is modeled along the surface of a 3D cell. B. The helix in A is projected onto a 2D plane. C. The helix in A becomes a straight line with an angle of θ when the cell is unrolled along its long axis. R is the radius of the cell, l is the pitch of the helix, and α is the tilting angle between line MN and the short axis of the cell, which can be measured from the projected image of the helix in A. D. Helices with variable pitches and lengths on flattened cell surfaces used to generate the helix models shown in Figure 3 for cells from A to D.
(0.89 MB TIF)

Figure S3 PALM band width measurement. The following example shows the procedure used to measure the width of each PALM band. A. The PALM image (i) of the cell expressing FtsZ-mEos2 shown in Figure 1C was first rotated to orient the cell's long axis parallel to the x-axis to generate the image in ii. The intensities of pixels along the y-axis at each position of the x-axis of this rotated image were averaged to generate the plot in (B). The intensity plot was then fitted with a Gaussian function to measure the width at the half maximum of the peak (pointed by two arrows), which was used as the width of the corresponding PALM band.
(0.39 MB TIF)

Figure S4 Dynamics of the Z-ring. Top row: Time-lapse epi-fluorescence imaging of cells expressing FtsZ-mEos2 (numbers are in minutes). Bottom row: Corresponding bright-field images of cells. Arrows in fluorescence images point to possible helical structures of the Z-ring. Bars, 500 nm.
(0.85 MB TIF)

Table S1 Comparison between PALM images and a helix model with a constant pitch.
(0.03 MB DOC)
