# Supplementary material for: In Vivo Structure of the E. coli FtsZ-ring Revealed by Photoactivated Localization Microscopy (PALM)
Source: PLoS One. 2010 Sep 13;5(9):e12680. doi: 10.1371/journal.pone.0012680 (PMC2938336; doi:10.1371/journal.pone.0012680)
Supplement: Text S1 — Construction and spatial resolution of PALM images. (0.02 MB DOCX) [file pone.0012680.s001.docx]

**Text S1. Construction and spatial resolution of PALM images**

**Molecular localization algorithm**

Molecular localization program was written in MATLAB (The MathWorks, Inc., Natick, MA). To find single FtsZ-mEo2 molecules in each image frame recorded with 570-nm excitation, the fluorescence intensity of the entire image was compared to an intensity threshold calculated from the running average of maximum intensity values across the entire PALM image stack. The running average was calculated using 1000 frames sampled at equal intervals through the PALM stack. All regions in a given PALM frame that had more than 3 adjacent pixels greater than the intensity threshold were considered to be FtsZ-mEos2 molecules and were fit using a nonlinear least squares algorithm (MATLAB function lsqcurvefit) to an assumed symmetric two-dimensional Gaussian function, which is an approximation of the point spread function (PSF):

 (1)

where (*x*_0_, *y*_0_) is the coordinate of the centroid position of the molecule, *A* is the fluorescence intensity at the centroid position, *B* is the background intensity, and *s* is the standard deviation of the Gaussian function. The fitted parameters *A*, *B*, *x*_0_, *y*_0_ and *s* were recorded for single FtsZ-mEos2 molecules. The majority of frames only had one single molecule of FtsZ-mEos2.

**Localization precision**

The theoretical localization precision is given by [1]:

 (2)

where *s* is the standard deviation of the PSF, *α* is the pixel size in the image, *N* is the total number of photons measured from the molecule, and *b* is the background noise. The number of photons a molecule emitted was converted from the EMCCD detector’s counts according to manufacturer’s specifications and camera settings. The pixel size of the camera is 16 µm, corresponding to an effective pixel size of 167 nm with a 96x magnification (60x from the objective and 1.6x from the microscope slider). We measured that the average photon number emitted from a single FtsZ-mEos2 molecule is 910 ± 690 photons (Figure S1B). Based on this formula, we calculated that our localization precision for all plotted FtsZ-mEos2 molecules was 14 ± 6 nm. These numbers agree well with published values of mEos2 [2].

**Construction of a PALM image**

PALM image rendering program was written in MATLAB (The MathWorks, Inc., Natick, MA). The position of each single FtsZ-mEos2 molecule in each image frame of the PALM sequence was determined using the algorithm described above. For fixed cells, if two molecules in successive frames were located less than three PALM pixels (45 nm) apart, we considered them to be the same molecule and the position of the molecule was recorded only once and the rest discarded. Only molecules with localization precisions smaller than 25nm (fixed cell) or 40nm (live cell) were plotted in the final PALM images. We plotted each molecule as a unit-area Gaussian function with *s* equal to the localization precision [3]. All Gaussian profiles were then superimposed together to generate the corresponding PALM image. The pixel size of PALM images was set at 15 nm. ImageJ software (NIH) was used to apply pseudocolor using the ‘red-hot’ lookup table.

**Spatial resolution**

Spatial resolution determines how far away two objects need to be in order to be spatially resolved from each other. We computed the theoretical spatial resolution in our PALM imaging using the following two methods. First, for single molecules that are localized to a precision of *n* nanometers, the theoretical spatial resolution is equal to the full width at half maximum (FWHM) of the replotted PSF, which is calculated by the formula FWHM=2.35*n*. Therefore, given the average localization precision at 14 nm, the minimal distance between two single FtsZ-mEos2 molecules needs to be 33 nm for them to be resolved.

Second, for protofilaments that consist of a mixture of wild-type FtsZ and FtsZ-mEos2 molecules, the Nyquist criterion states that the ability to distinguish two such protofilaments on a 2D PALM image is directly related to the labeling density of the protofilaments such that

 (3)

where *d* is the desired spatial resolution in nm and *ρ* is the density of FtsZ-mEos2 molecules that can be resolved to *d* or better in molecules/nm^2^ [4-6]. We measured the average labeling density for each cell by dividing the total number of detected FtsZ-mEos2 molecules by the corresponding occupied area in the PALM image (ranged from 0.003 to 0.015 molecules/nm^2^), and calculated an average spatial resolution to be 28 ± 7 nm. The average spatial resolution defined by Nyquist criterion is indistinguishable from the one calculated from the localization precision of single FtsZ-mEos2 molecules, indicating that here the spatial resolution was not limited by the labeling density of the Z-ring.

The above value is the upper bound of achievable spatial resolution. We calculated the actual spatial resolution by determining the displacement distribution of single FtsZ-mEos2 molecules that lasted more than one frame in PALM imaging sequences. If the displacement between the fitted positions of two molecules in two consecutive frames was less than 167 nm, we treated these two molecules as the same molecule. Therefore, the positions generated by the same molecule in consecutive frames reflected the error in the position determination of single molecules, i.e., the actual spatial resolution of PALM imaging. We plotted the distribution of the displacement of all these molecules and found the distribution peaked at 33.9 nm (Figure S1C). Therefore, the actual spatial resolution was ~35 nm.

**References**

1. Thompson RE, Larson DR, Webb WW (2002) Precise nanometer localization analysis for individual fluorescent probes. Biophys J 82: 2775-2783.

2. McKinney SA, Murphy CS, Hazelwood KL, Davidson MW, Looger LL (2009) A bright and photostable photoconvertible fluorescent protein. Nat Methods 6: 131-133.

3. Betzig E, Patterson GH, Sougrat R, Lindwasser OW, Olenych S, et al. (2006) Imaging intracellular fluorescent proteins at nanometer resolution. Science 313: 1642-1645.

4. Shannon C (1949) Communication in the presence of noise. Proc Inst Radio Eng 37: 10-21.

5. Shroff H, White H, Betzig E (2008) Photoactivated localization microscopy (PALM) of adhesion complexes. Curr Protoc Cell Biol Chapter 4: Unit 4 21.

6. Greenfield D, McEvoy AL, Shroff H, Crooks GE, Wingreen NS, et al. (2009) Self-organization of the Escherichia coli chemotaxis network imaged with super-resolution light microscopy. PLoS Biol 7: e1000137.
