# Supplementary material for: In Vivo Structure of the E. coli FtsZ-ring Revealed by Photoactivated Localization Microscopy (PALM)
Source: PLoS One. 2010 Sep 13;5(9):e12680. doi: 10.1371/journal.pone.0012680 (PMC2938336; doi:10.1371/journal.pone.0012680)
Supplement: Text S2 — Modeling of 3D helices and simulations of PALM images. (0.02 MB DOCX) [file pone.0012680.s002.docx]

**Text S2. Modeling of 3D helices and simulations of PALM images**

**Simulation of helices with variable length and pitch**

Figure S2A depicts a helix with a radius, *R,* and constant pitch, *l,* wrapping the cylindrical surface of a typical *E. coli* cells 1.5 turns. Based on our single cell measurements, *R* typically equals 0.5 µm. When projected onto a 2D imaging plane along the long axis of the cell (Figure S2B), this 3D helix results in two parallel lines (red solid lines) that are separated by a distance equal to the pitch (*l)* and are tilted at an angle (*α)* with respect to the short axis of the cell. *α* is directly related to *l* in that tan(*α*) = *l*/4*R.* The larger the tilting angle, the larger the helical pitch, and hence the wider the separation between the two parallel bands. Therefore, by measuring *α* from the 2D PALM image we can calculate what the pitch of the helix should be (*l*’). We then compare this calculated pitch (*l*’) with the measured separation between the two parallel bands (*l)* to check if the two bands indeed result from the projection of a helix with constant pitch (*l)*. Table S1 lists the measured tilting angles (*α)*, cell radius (*R)*, measured separation between the two bands (*l*) and the calculated pitch (*l*’) for the two cells shown in Figure 3D and 3E. We found that in each case *l*’ is significantly different from *l*, indicating that the parallel bands did not result from a continuous helix with constant helical pitch.

To find out if a helix with variable pitch indeed could give rise to the observed pattern of parallel bands for cells shown in Figure 3D and 3E, we modeled such a helix inside the cell and simulated the resulting 2D PALM images using custom code in MATLAB. A helix with a constant pitch along the cell’s inner surface would turn into a straight line if the cell is unrolled along its long axis (Figure S2C). This straight line can be described by the equation *y’* = (*l* / 2*πR*) · *x’*. The angle of the line with respect to the short axis (*θ)* of the cell is related to the pitch in the form of tan(*θ)* = *l* / 2*πR*. If *θ* is equal to zero, the line will be perpendicular to the cell’s long axis, leading to a ring if the cell is rolled up. If *θ* is greater than zero and varies along the length, the resulting helix will have variable pitch, leading to a curve instead of a straight line on the flattened cell surface. Therefore, we used a curve that consists of short straight lines connected by short smooth arcs to model the helix. The length and pitch of each segment and the length and diameter of the cell were measured from the corresponding PALM image.

After generating such a helix model inside the cell, we simulated the resulting PALM image of such a structure. To simplify the simulation, we assumed that the helix was a single layer flat ribbon that was seven FtsZ protofilaments thick [1] (Note that here we focus on the conformation of the Z-ring so the arrangement of protofilaments inside the Z-ring is of less importance). The spacing between each neighboring filament was 9 nm and each FtsZ monomer in the filament was spaced 5 nm from the next one in the longitudinal direction [2]. We then simulated the PALM imaging process by randomly “activating” FtsZ molecules inside the helix one at a time and projected its image along the long axis of the cell onto a 2D plane. Specifically, an FtsZ molecule with a coordinate of (*x, y, z*) inside the helix was randomly selected and its projected position (*x’, y’*) on the 2D plane was determined. Next, we simulated the fluorescence image of this molecule on the 2D plane using a Gaussian PSF function. This PSF function was then modified by taking into account the following factors: (1) the number of photons described by this PSF followed a normal distribution; (2) the width of the PSF (defocusing effect) was calculated using the distance of the FtsZ molecule away from the focal plane of the objective lens [3]; and (3) background noise, including photon shot noise estimated based on real imaging conditions, was added to the PSF. We then fit the simulated fluorescence image of the molecule on the 2D plane using the molecular localization algorithm, generating the corresponding centroid position (x”, y”) and the associated standard deviation *σ_xy_* of the molecule. Using the *σ_xy_* and simulated photon numbers we calculated the localization precision of this molecule and plotted its image as a unit-Gaussian PSF with its standard deviation equal to the localization precision. This process was then repeated thousands of times until ~25% of FtsZ molecules inside the helical structure were selected and localized. The final resulting image, the simulated PALM image, was then compared with the original image to verify if the separation and tilting angle of the parallel bands matched those measured from the original image. If not, *θ* was varied and the above process repeated until the resulting simulated PALM matched reasonably well with the original PALM image. Using this procedure we simulated PALM images for cells shown in Figure 3A-D. The corresponding helices on a flattened cell surfaces for these cells were shown in Figure S2D.

**References**

1. Stricker J, Maddox P, Salmon ED, Erickson HP (2002) Rapid assembly dynamics of the Escherichia coli FtsZ-ring demonstrated by fluorescence recovery after photobleaching. Proc Natl Acad Sci U S A 99: 3171-3175. Epub 2002 Feb 3119.

2. Li Z, Trimble MJ, Brun YV, Jensen GJ (2007) The structure of FtsZ filaments in vivo suggests a force-generating role in cell division. Embo J 26: 4694-4708.

3. van Oijen AM, Köhler J, Schmidt J, Müller M, Brakenhoff GJ (1998) 3-Dimensional super-resolution by spectrally selective imaging. Chem Phys Lett 292: 183-187.
