# Supplementary figures and images for: In Vivo Structure of the E. coli FtsZ-ring Revealed by Photoactivated Localization Microscopy (PALM)
Source: PLoS One. 2010 Sep 13;5(9):e12680. doi: 10.1371/journal.pone.0012680 (PMC2938336; doi:10.1371/journal.pone.0012680)

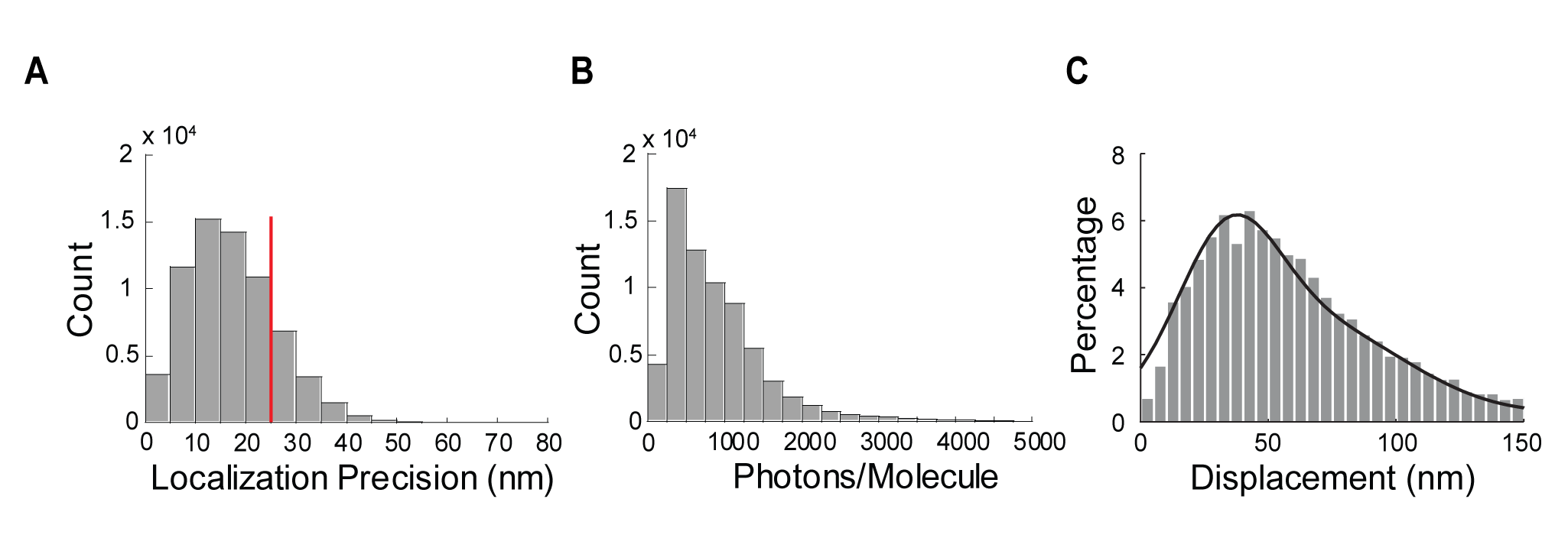

Supplement: Figure S1 — Localization precision and spatial resolution of PALM images. A. Histogram of all the localization precisions for all the molecules detected from both the B/r A and BL21(DE3)pLysS fixed samples (mean = 17 nm), As indicated by the red line, only molecules possessing a localization precision of 25 nm or better were used to construct fixed PALM images. B. Histogram of total number of photons detected for all the FtsZ-mEos2 molecules detected in fixed B/r A and BL21 samples (mean = 910 photons). C. Displacement distribution of single FtsZ-mEos2 molecules that lasted more than one frame in PALM imaging sequences. The positions generated by the same molecule in consecutive frames reflect the error in the position determination of single molecules, i.e., the actual spatial resolution of PALM imaging. The distribution of the displacement was fit with the sum of two normal distributions with the major peak at 33.9 nm. (0.23 MB TIF) [file pone.0012680.s003.tif]

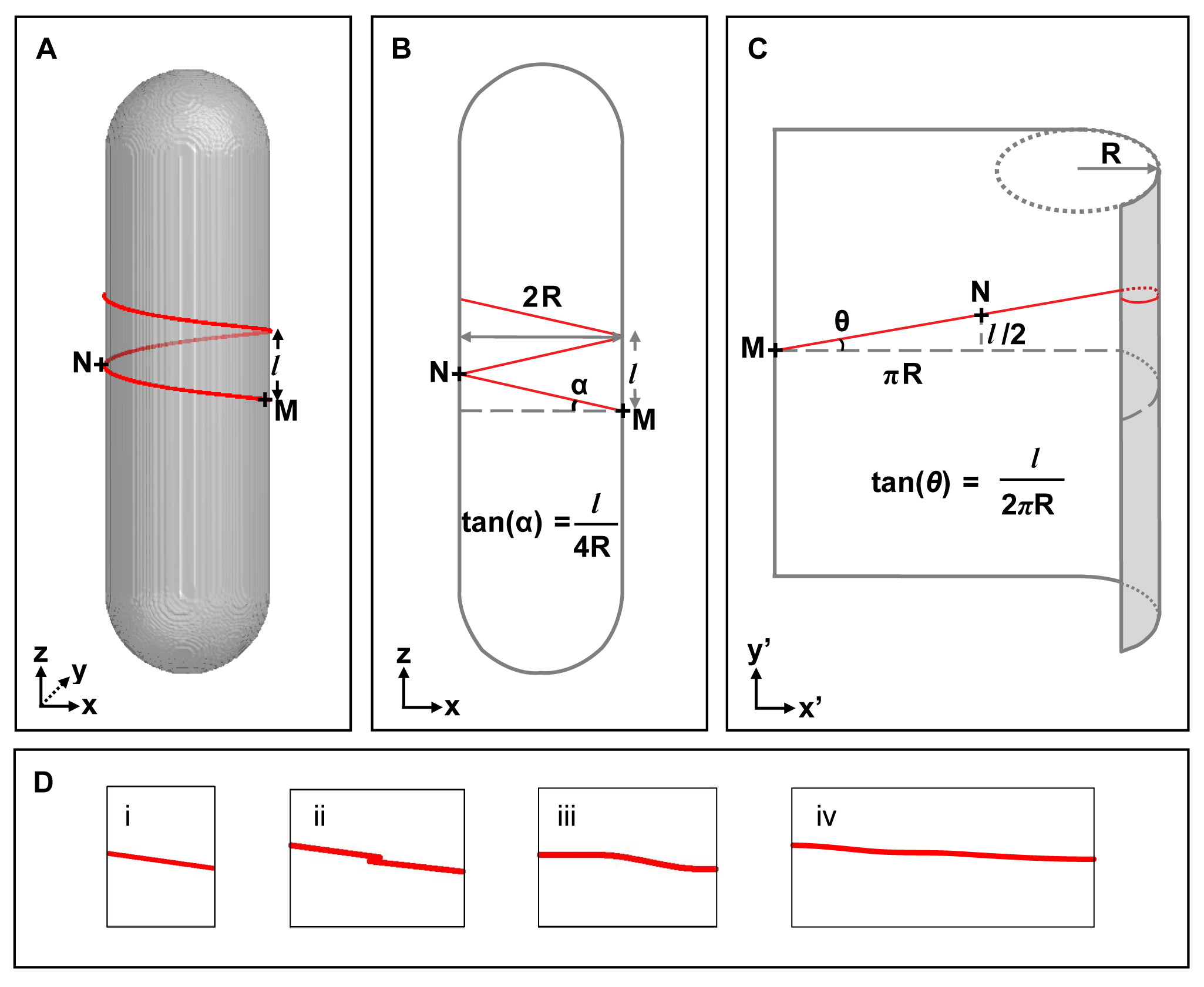

Supplement: Figure S2 — The helical model used to simulate the PALM images in Figure 3. A. A helix (red) is modeled along the surface of a 3D cell. B. The helix in A is projected onto a 2D plane. C. The helix in A becomes a straight line with an angle of θ when the cell is unrolled along its long axis. R is the radius of the cell, l is the pitch of the helix, and α is the tilting angle between line MN and the short axis of the cell, which can be measured from the projected image of the helix in A. D. Helices with variable pitches and lengths on flattened cell surfaces used to generate the helix models shown in Figure 3 for cells from A to D. (0.89 MB TIF) [file pone.0012680.s004.tif]

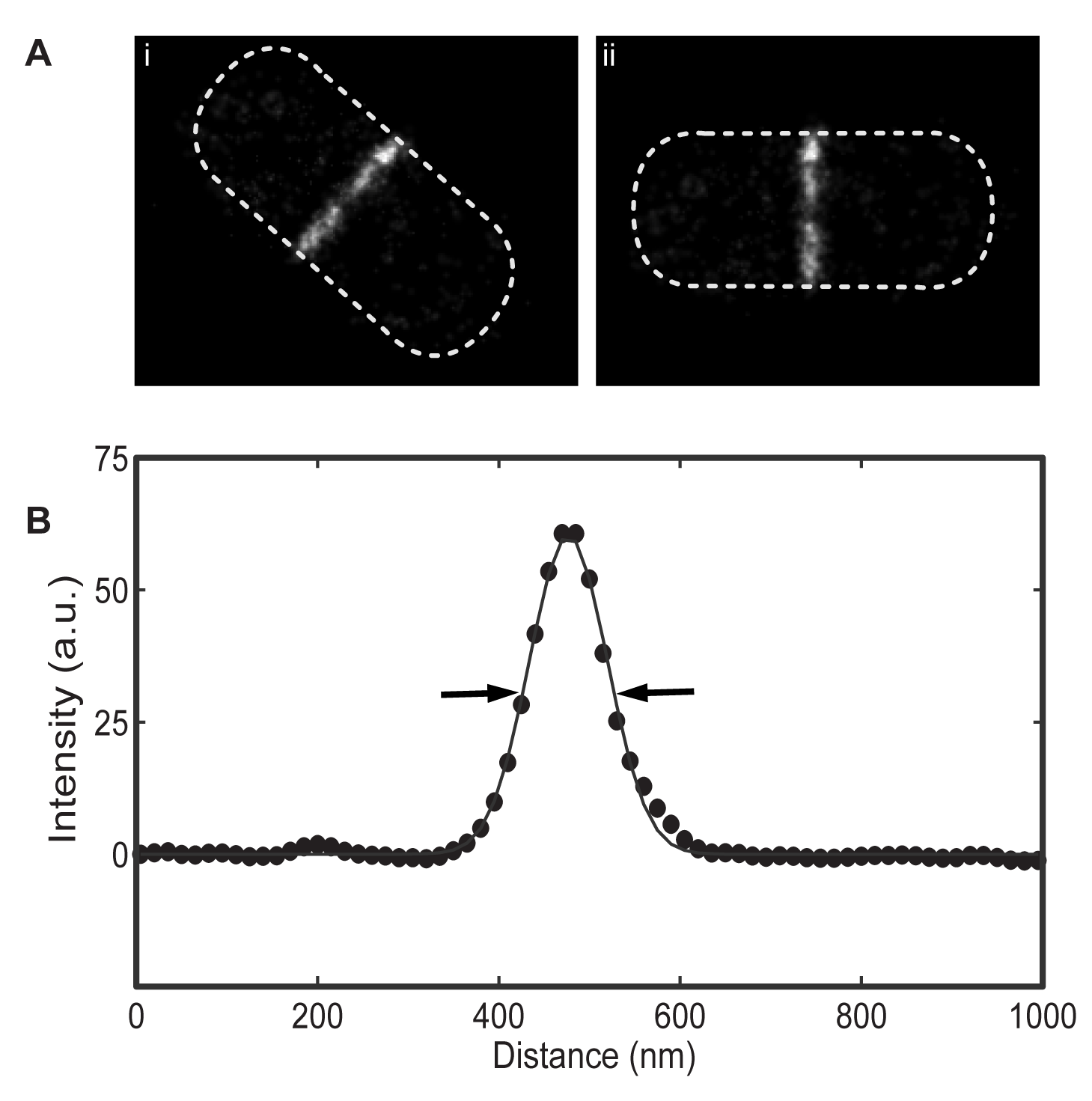

Supplement: Figure S3 — PALM band width measurement. The following example shows the procedure used to measure the width of each PALM band. A. The PALM image (i) of the cell expressing FtsZ-mEos2 shown in Figure 1C was first rotated to orient the cell's long axis parallel to the x-axis to generate the image in ii. The intensities of pixels along the y-axis at each position of the x-axis of this rotated image were averaged to generate the plot in (B). The intensity plot was then fitted with a Gaussian function to measure the width at the half maximum of the peak (pointed by two arrows), which was used as the width of the corresponding PALM band. (0.39 MB TIF) [file pone.0012680.s005.tif]

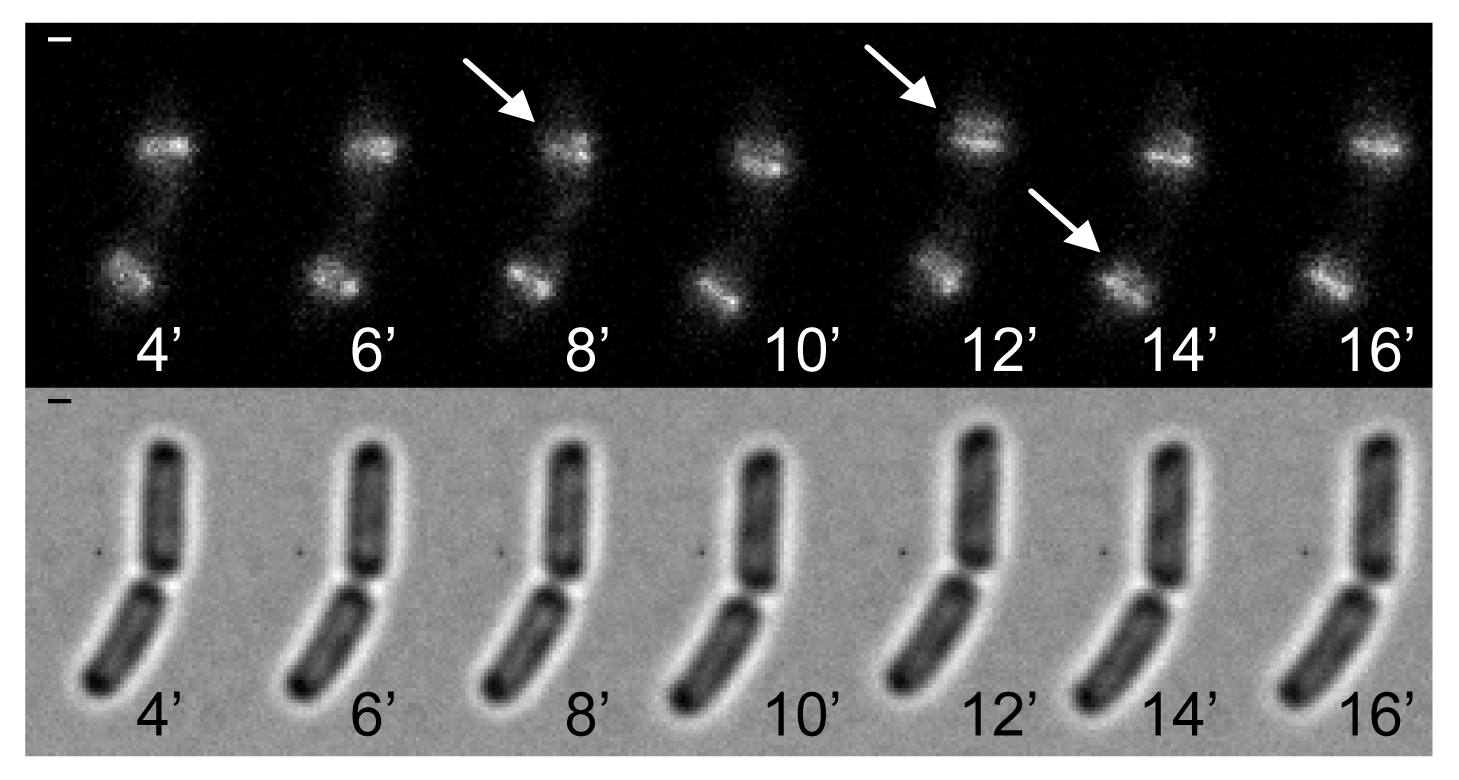

Supplement: Figure S4 — Dynamics of the Z-ring. Top row: Time-lapse epi-fluorescence imaging of cells expressing FtsZ-mEos2 (numbers are in minutes). Bottom row: Corresponding bright-field images of cells. Arrows in fluorescence images point to possible helical structures of the Z-ring. Bars, 500 nm. (0.85 MB TIF) [file pone.0012680.s006.tif]
