# Supplementary material for: In Vivo Structure of the E. coli FtsZ-ring Revealed by Photoactivated Localization Microscopy (PALM)
Source: PLoS One. 2010 Sep 13;5(9):e12680. doi: 10.1371/journal.pone.0012680 (PMC2938336; doi:10.1371/journal.pone.0012680)
Supplement: Table S1 — Comparison between PALM images and a helix model with a constant pitch. (0.03 MB DOC) [file pone.0012680.s007.doc]

| Table S1: **Comparison between PALM images and a helix model with a constant pitch**. Cells C and D of Figure 3 were used for the comparison. Listed are the tilting angle relative to the cell’s short axes for PALM bands (*α)*, cell diameter (2*R)* and the pitch (*l*) between PALM bands. The expected pitch (*l*’) is calculated from a constant-pitch helix model using *α* according to the equation from Figure S2B. *l’* is significantly different from *l*, indicating that the parallel bands did not result from a helix with a constant pitch. | | | | |
| --- | --- | --- | --- | --- |
| Cell | *α* (degree) | 2*R* (nm) | *l* (nm) | *l’* (nm) |
| C | 0.4 | 1035 | 330 | 14 |
| D | 1.4  . | 960 | 195/360 | 47 |
